# The efficacy and tolerability of latency-reversing agents in reactivating the HIV-1 reservoir in clinical studies: a systematic review

**DOI:** 10.1016/j.jve.2023.100342

**Published:** 2023-08-19

**Authors:** Quinten Debrabander, Kathryn S. Hensley, Christina K. Psomas, Wichor Bramer, Tokameh Mahmoudi, Berend J. van Welzen, Annelies Verbon, Casper Rokx

**Affiliations:** a Department of Internal Medicine and Infectious Diseases, University Medical Centre Utrecht, Mailbox 85500, 3508GA, Utrecht, the Netherlands; b Department of Internal Medicine, Section Infectious Diseases, And Department of Medical Microbiology and Infectious Diseases, Erasmus MC, Erasmus University Medical Centre, P.O. Box 2040, 3000CA, Rotterdam, the Netherlands; c Department of Infectious Diseases and Internal Medicine, European Hospital, Marseille, France; d Medical Library, Erasmus MC, Erasmus University Medical Centre, P.O. Box 2040, 3000CA, Rotterdam, the Netherlands; e Department of Biochemistry, Erasmus MC, Erasmus University Medical Center, P.O. Box 2040, 3000CA, Rotterdam, the Netherlands; f Department of Pathology, Erasmus MC, Erasmus University Medical Center, P.O. Box 2040, 3000CA, Rotterdam, the Netherlands; g Department of Urology, Erasmus MC, Erasmus University Medical Center, P.O. Box 2040, 3000CA, Rotterdam, the Netherlands

**Keywords:** Human immunodeficiency virus, Latency reactivation, Virus latency, Virus activation, Interleukins, Systematic review

## Abstract

**Introduction:**

Understanding the clinical potency of latency-reversing agents (LRAs) on the HIV-1 reservoir is useful to deploy future strategies. This systematic review evaluated the effects of LRAs in human intervention studies.

**Methods:**

A literature search was performed using medical databases focusing on studies with adults living with HIV-1 receiving LRAs. Eligibility criteria required participants from prospective clinical studies, a studied compound hypothesised as LRA, and reactivation or tolerability assessments. Relevant demographical data, LRA reactivation capacity, reservoir size, and adverse events were extracted. A study quality assessment with analysis of bias was performed by RoB 2 and ROBINS-I tools. The primary endpoints were HIV-1 reservoir reactivation after LRA treatment quantified by cell-associated unspliced HIV-1 RNA, and LRA tolerability defined by adverse events. Secondary outcomes were reservoir size and the effect of LRAs on analytical treatment interruption (ATI) duration.

**Results:**

After excluding duplicates, 5182 publications were screened. In total 45 publications fulfilled eligibility criteria including 26 intervention studies and 16 randomised trials. The risk of bias was evaluated as high. Chromatin modulators were the main investigated LRA class in 24 studies. Participants were mostly males (90.1%). Where reported, HIV-1 subtype B was most frequently observed. Reactivation after LRA treatment occurred in 78% of studies and was observed with nearly all chromatin modulators. When measured, reactivation mostly occurred within 24 h after treatment initiation. Combination LRA strategies have been infrequently studied and were without synergistic reactivation. Adverse events, where reported, were mostly low grade, yet occurred frequently. Seven studies had individuals who discontinued LRAs for related adverse events. The reservoir size was assessed by HIV-1 DNA in 80% of studies. A small decrease in reservoir was observed in three studies on immune checkpoint inhibitors and the histone deacetylase inhibitors romidepsin and chidamide. No clear effect of LRAs on ATI duration was observed.

**Conclusion:**

This systematic review provides a summary of the reactivation of LRAs used in current clinical trials whilst highlighting the importance of pharmacovigilance. Highly heterogeneous study designs and underrepresentation of relevant patient groups are to be considered when interpreting these results. The observed reactivation did not lead to cure or a significant reduction in the size of the reservoir. Finding more effective LRAs by including well-designed studies are needed to define the required reactivation level to reduce the HIV-1 reservoir.

## 1 Introduction

Despite many advances in the treatment of the human immunodeficiency virus type 1 (HIV-1) infection, people living with HIV-1 still require lifelong antiretroviral therapy (ART). Cessation of therapy results in a rebound of plasma viremia and, ultimately, in disease progression and death. This process is driven by viral reactivation from the latently infected reservoir, primarily consisting of memory CD4^+^ T-cells, which have HIV-1 viral genome integrated into their DNA.^1^ In these cells, the replication-competent proviruses are largely repressed by mechanisms that prevent transcription and immune clearance. This latent reservoir currently remains the greatest barrier to cure HIV-1.^2^

Although a cure was achieved in a few people living with HIV-1 by means of allogeneic stem cell transplantation, the high procedural complication risk makes this an unfeasible option for large-scale cure efforts.^3^, ^4^, ^5^ This observation emphasises the importance of alternative cure strategies. Currently, one of the approaches towards a feasible HIV-1 cure is the ‘shock and kill’ or ‘kick and kill’ strategy.^6^ In the former strategy, drugs called latency-reversing agents (LRAs) reactivate viral transcription in the latently infected cells within the reservoir. LRAs with demonstrated potency in cells from people living with HIV-1 have been hypothesised to reactivate latency through different mechanisms of action.^7^ One group of LRAs target chromatin regulation and alter the chromatin environment allowing for proviral transcription, such as histone deacetylase inhibitors (HDACis). A second group works on a more downstream level, activating transcription at the HIV-1 promotor through compounds such as some toll-like receptor (TLR) agonists or protein kinase C (PKC) agonists. Other mechanisms involve targeting the immune system with compounds that have also shown proviral reactivation such as immune checkpoint inhibitors, interleukins, or vaccinations. Irrespective of their mechanism of action, LRAs induce viral antigen production in reservoir cells which is anticipated to trigger immune-mediated infected cell killing by itself or does so when combined with compounds that induce cell-death of reactivated cells, the outcome of which is a decrease in the viral reservoir and, theoretically, a cure.

The diverse mechanisms of action of different LRAs have been described in overviews of preclinical study findings which have focused less on the *in vivo* LRA efficacy.^7^, ^8^, ^9^ In order to deploy successful future LRA strategies, there is a need for a deeper understanding of the clinical efficacy and tolerability of the current LRAs, as well as the identification of knowledge gaps that need to be addressed in future trials.

Here, we have systematically reviewed published clinical studies on the efficacy and tolerability of LRAs in people living with HIV-1. The results of this review can be used for future trial designs, including sampling, selection of the study populations, and LRAs capable of most potently reactivating the reservoir with the aim to promote reservoir decay and ultimately a cure.

## 2 Materials and methods

### 2.1 Search strategy and study selection

This systematic review is reported using the guidelines of Preferred Reporting Items for Systematic Reviews and Meta-Analyses (PRISMA) 2020.^10^ The PRISMA checklist is available in Appendix 1. The trial protocol was registered on the International Prospective Register of Systematic Reviews (PROSPERO) record ID CRD42022341021.

We HAVE performed a literature search in Embase.com, MEDLINE ALL in Ovid, Web of Science Core Collection, and the Cochrane Central Register of Controlled Trials via Wiley, up to January 9, 2023, for adult people living with HIV-1 receiving LRAs *in vivo* and enrolled in a prospective clinical study. The search string developed by an experienced information specialist (WMB) combined terms for HIV-1, LRAs, and the currently known classes of LRAs, as well as individual compounds. The full search string for all databases can be found in Appendix 2. After removal of duplicates in EndNote using the method as described by Bramer et al.^11^, two reviewers (QD, KSH) independently screened all studies for inclusion using Rayyan.^12^ In case of disparities a third reviewer decided on inclusion (CR). References of included studies were evaluated on potential missed studies.

### 2.2 Eligibility criteria

Criteria for study inclusion in the review were predefined as being human intervention trials, prospective cohort studies, prospective case reports or series, on LRAs in people living with HIV-1, including post hoc analyses of these studies. Latency was defined as the presence in long-lived human cells of a viral genome that is transcriptionally silenced but retained the ability to reactivate proviral transcription.^13^ If the authors hypothesised that the studied treatment reactivated proviral transcription in reservoir cells, then the treatment was considered an LRA. Viral transcriptional activity by cell-associated (CA) unspliced (US) HIV-1 RNA or adverse events (AEs) had to be reported as outcomes.^6^ Retrospective case reports or series, and unpublished non-peer reviewed conference presentations were excluded, as well as reviews.

### 2.3 Quality assessment and data extraction

A risk of bias analysis was performed using the Revised Cochrane risk-of-bias tool for randomised trials (RoB 2) and the Risk of Bias in Non-randomised Studies of Interventions (ROBINS-I) tool for observational studies.^14^^,^^15^ We analysed the risk of bias with the R package ‘robvis’.^16^

Data was extracted by two reviewers (QD, KSH) with a standardised template using Microsoft Excel and included study design, authors, publication year, study location, participant demographics (age, sex, ethnicity), CD4^+^ and CD8^+^ T cell count (nadir and at the start of the investigational treatment defined as baseline), plasma HIV-1 RNA (zenith and at baseline), characteristics of HIV-1 infection (HIV-1 subtype, Fiebig stage at ART initiation, ART history) and the studied intervention. LRAs were categorised according to their mechanism of action: chromatin modulators, activators of transcription, immune checkpoint inhibition, interleukins or interleukin agonists, vaccines, and any other mechanisms.^7^ Outcome data collected in all selected studies were CA US HIV-1 RNA measurements, adverse events (number and grading) including discontinuation rates due to adverse events and serious adverse events (SAEs), and, if available, reservoir size measurements [total HIV-1 DNA, quantitative viral outgrowth assay (QVOA) and Tat/Rev Induced Limiting Dilution Assay (TILDA)].

### 2.4 Endpoints and data synthesis

The primary endpoints were 1) HIV-1 reservoir reactivity after LRA treatment, determined by CA US HIV-1 RNA and 2) LRA tolerability. The CA US HIV-1 RNA fold change from baseline was focused on the first CA US HIV-1 RNA measurement after the initial LRA dose, as well as the maximal change observed. The expected significant heterogeneous study designs by LRA treatment duration and sampling strategy prevented choosing one timepoint to assess endpoints. Study designs and measurement timepoints were evaluated on heterogeneity and the possibility to compare endpoints between studies. The secondary endpoints were the size of the HIV-1 reservoir and, in studies that included analytical treatment interruptions (ATI), the effect of LRAs on the total duration of the ATI. Results were synthesised following the Synthesis Without Meta-analysis (SWiM) guidelines.^17^

## 3 Results

### 3.1 Characteristics of included studies

**Fig. 1:**
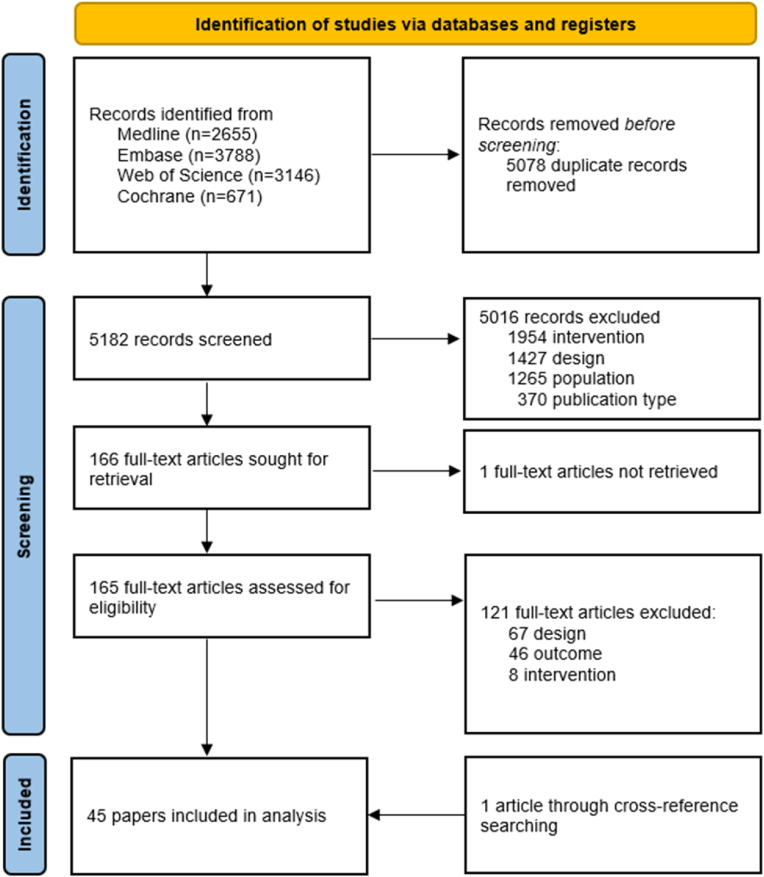
Flow chart of the study selection (1.5-column fitting image).

Overall, we have identified 10,260 articles of interest (Fig. 1). Of these, 5078 duplicates were removed, and of 5182 articles title and abstract were screened. Most studies (5016) were ineligible because the interventions were not hypothesised to act as an LRA by the authors, were preclinical studies or reviews, did not enrol people with HIV-1, were conference abstracts, editorials, or not written in English. A total of 45 manuscripts were included in the final analysis after full-text screening. Study designs included non-randomised studies of interventions (NRSIs) (57.8%), randomised controlled trials (RCTs) (35.6%), case series (4.4%), and one post-hoc analysis (2.2%) (Table 1). Twenty-two published studies examined the effect of LRAs from the chromatin modulation class, mostly HDACis, including 9 using vorinostat and 6 romidepsin. A sum of 975 participants were included, with 490 participating in a trial that investigated chromatin modulators. A total of 38 studies were from North America or Europe and enrolled a median of 16 participants (IQR 10–30).

**Table 1:** Study characteristics of published clinical studies on people with HIV-1 exposed to latency-reversing agents grouped according to their mechanism of action.

Author	Publication year	Study arms	Design	Sample size	Study period	Countries involved
Chromatin modulators						
Histone deacetylase inhibitors (HDACis)						
Archin et al.^18^	2008	- Valproic acid	NRSI	12	NI	US
Archin et al.^19^	2010	- Valproic acid^a^ - Valproic acid + Raltegravir - Valproic acid + Enfuvirtide - Enfuvirtide	NRSI	12	NI	US
Routy et al.^20^	2012	- Valproic acid (16 weeks) - Valproic acid (32 weeks)	RCT	56	2006–2009	Canada
Archin et al.^21^	2012	- Vorinostat	NRSI	8	NI	US
Archin et al.^22^	2014	- Vorinostat	NRSI	5	NI	US
Elliott et al.^23^	2014	- Vorinostat	NRSI	20	NI	US
Archin et al.^24^	2017	- Vorinostat	NRSI	16	NI	US
Fidler et al.^25^	2020	- Vorinostat + ChAdV63.HIVconsv + MVA.HIVconsv boost vaccine - Control	RCT	60	2015–2017	UK
Gay et al.^26^	2020	- Vorinostat + AGS-004	NRSI	5	NI	US
Kroon et al.^27^	2020	- Vorinostat + hydroxychloroquine + maraviroc - Control	RCT	15	2015	Thailand
Gay et al.^28^	2022	- Vorinostat + VRC07-523LS	NRSI	8	NI	US
Scully et al.^29^	2022	- Tamoxifen + vorinostat - Vorinostat	RCT	31	2018	US
Rasmussen et al.^30^	2014	- Panobinostat	NRSI	15	2012–2014	Denmark
Sogaard et al.^31^	2015	- Romidepsin	NRSI	6	2014	Denmark
Leth et al.^32^	2016	- Romidepsin + Vacc-4x + rhuGM-CSF	NRSI	20	2014–2015	Denmark
Mothe et al.^33^	2020	- Romidepsin + MVA.HIVconsv vaccine^b^	NRSI	15	2016–2017	Spain
McMahon et al.^34^	2021	- Romidepsin 0.5 mg/m^2^ × 1 - Romidepsin 2.0 mg/m^2^ × 1 - Romidepsin 5.0 mg/m^2^ × 1 - Placebo (×1) - Romidepsin 5.0 mg/m^2^ × 4 - Placebo (×4)	RCT	59	NI	US
Gruell et al.^35^	2022	- Romidepsin - Romidepsin + 3BNC117	RCT	20	2017–2018	US, Denmark, Germany
Gunst et al.^36^	2022	- Romidepsin - 3BNC117 - Romidepsin + 3BNC117 - Control	RCT	59	2017–2020	Denmark, UK
Li et al.^37^	2020	- Chidamide	NRSI	7	NI	China
***IKAROS Family Zinc Finger 1 protein (IKZF1) degradation***						
Liu et al.^38^	2022	- Lenalidomide	NRSI	13	2019–2020	China
***BRG-1-associated factors complex inhibitors (BAFis)***						
Prins et al.^39^	2023	- Pyrimethamine - Valproic acid - Valproic acid + pyrimethamine - Control	RCT	28	2018–2020	Netherlands
**Transcription activators**						
***Phosphatase and tensin homolog (PTEN) dysregulation***						
Spivak et al.^40^	2013	- Disulfiram	NRSI	16	NI	US
Elliot et al.^41^	2015	- Disulfiram (500 mg) - Disulfiram (1000 mg) - Disulfiram (2000 mg)	NRSI	30	2013–2014	US, Australia
McMahon et al.^42^	2022	- Disulfiram + vorinostat	NRSI	2	NI	Australia
***Toll-like receptor (TLR) agonists***						
Vibholm et al.^43^	2017	- MGN1703	NRSI	15	2015	Denmark
Vibholm et al.^44^	2019	- MGN1703	NRSI	12	2016–2017	Denmark
Saxena et al.^45^	2019	- Poly-ICLC - Placebo	RCT	15	NI	US
Riddler et al.^46^	2020	- Vesatolimod 1 mg - Vesatolimod 2 mg - Vesatolimod 4 mg - Vesatolimod 6 mg - Vesatolimod 8 mg - Vesatolimod 10/12 mg - Placebo	RCT	48	2015–2018	US
***Non-canonical NFKb agonists***						
Lafeuillade et al.^47^	2014	- Maraviroc + raltegravir - Control	RCT	22	NI	France
Madrid-Elena et al.^48^	2018	- Maraviroc	NRSI	20	2012–2015	Spain
Lopez-Huertas et al.^49^	2020	- Maraviroc	Post-hoc analysis	3	2008–2015	Spain
***Protein kinase C (PKC) agonists***						
Gutiérrez et al.^50^	2016	- Bryostatin (20μg/m^2^) - Bryostatin (10μg/m^2^) - Placebo	RCT	12	2014–2015	Spain
**Interleukins and interleukin (IL) agonists**						
Stellbrink et al.^51^	2002	- IL-2 - Control	RCT	56	NI	Germany
Katlama et al.^52^	2016	- IL-7 + faltegravir + maraviroc - Raltegravir + Maraviroc	RCT	29	2010–2011	France
Miller et al.^53^	2022	- N-803 (IL-15 agonist) (0.3mcg/kg) - N-803 (1.0mcg/kg) - N-803 (3.0mcg/kg) - N-803 (6.0mcg/kg)	NRSI	16	2015–2019	US
**Immune checkpoint (IC) inhibitors**						
Wightman et al.^54^	2015	- Ipilimumab	Case report^e^	1	NI	Australia
Lau et al.^55^	2021	- Avelumab - Ipilimumab + nivolumab	Case series^e^	3	NI	Australia
Rasmussen et al.^56^	2021	- Nivolumab - Nivolumab + ipilimumab	NRSI	40	2016–2019	US
Uldrick et al.^57^	2022	- Pembrolizumab	NRSI	32	NI	US
**Vaccines**						
Achenbach et al.^58^	2015	- ART intensification^c^ + HIV DNA vaccine + rAD5 boost - ART intensification^c^	RCT	28	2010–2011	US
Yek et al.^59^	2016	- Vaccination schedule^d^ - Placebo	RCT	26	NI	Spain
Christensen-Quick et al.^60^	2018	- Influenza vaccine (Fluarix)	NRSI	7	NI	US
Stevenson et al.^61^	2022	- BNT162b2 mRNA - mRNA-1273	NRSI	35	NI	Canada, US
**Other**						
Cummins et al.^62^	2021	- Ixazomib	NRSI	17	2017–2019	US

a Three participants from the Archin 2008 study, who received 16 weeks of VPA in the 2008 study and received an additional 16 weeks of VPA in this study.

b BCN02 study, follow-up of BCN01 where participants received ChAdV63. HIVconsv and MVA. HIVconsv vaccine.

c Raltegravir + Maraviroc.

d Hepatitis A, Hepatitis B, Influenza, Pneumococcal, Tetanus-diphtheria, Varicella, Measles-Mumps-Rubella.

e Prospective. US: United States, UK: United Kingdom, NRSI: non-randomised study of intervention, RCT: randomised controlled trial, rHuGM-CSF: recombinant human granulocyte-macrophage colony-stimulating factor, NI: no information, IL: interleukin, VPA: valproic acid.

Most participants were male (90.1%, Table S1), and 15 studies exclusively included male participants. Median age was 49 years (IQR 44–53) and, when reported, participants were mostly white (71.5%). Median CD4^+^ T cell count at study enrolment was 661 cells/mm^3^ (IQR 582–720) after a median of 7.5 years (IQR 3.6–9.9) of ART including 4.4 years (IQR 2.6–6.6) with plasma viral suppression. Only 13.3% of all studies reported HIV-1 subtype, and 62.8% of participants in these studies were living with HIV-1 subtype B. Three studies exclusively included people who initiated ART during acute HIV-1 infection,^25^^,^^27^^,^^33^ 7 studies those who had initiated ART whilst chronically infected^34^^,^^44^^,^^47^^,^^52^^,^^54^^,^^58^^,^^60^ and the other studies included participants regardless of the timing of ART initiation. Two studies started LRA treatment at the time of ART initiation.^36^^,^^51^

### 3.2 HIV-1 reservoir reactivity

Thirty-six studies reported CA US HIV-1 RNA, with 77.8% reporting a fold change increase at any given timepoint during or after LRA treatment (Table 2). Of the studies reporting an increase, most used chromatin modulators (19 studies) of which 89.5% HDACis. Significant increases from baseline CA US HIV-1 RNA were found in all HDACis except for valproic acid monotherapy.^39^ Of the eleven studies with transcription activators, CA US HIV-1 RNA increases were found in five studies,^34^^,^^41^^,^^44^^,^^48^^,^^49^ with maraviroc^48^ and disulfiram^41^ having the best evidence for their effect. Furthermore, all four immune checkpoint inhibitors^54^, ^55^, ^56^, ^57^ and two vaccine studies^59^^,^^60^ demonstrated CA US HIV-1 RNA increases. No information regarding reactivation was provided in studies involving interleukins (ILs). LRAs were combined with other therapies in six studies (vorinostat with maraviroc, vorinostat with tamoxifen, pyrimethamine with valproic acid, vorinostat with disulfiram, IL-7 with maraviroc, and nivolumab with ipilimumab).^27^^,^^39^^,^^42^^,^^52^^,^^55^^,^^56^ Increase in CA US HIV-1 RNA was observed in all combinations except IL-7 with maraviroc, but none exhibited synergy between LRAs.

**Table 2:**
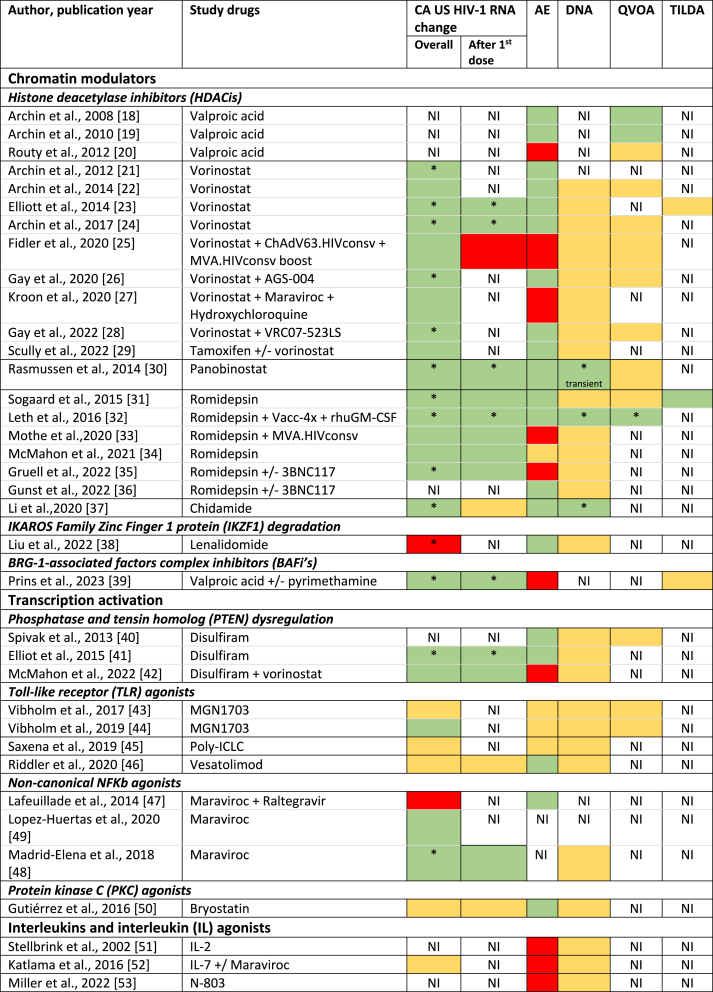

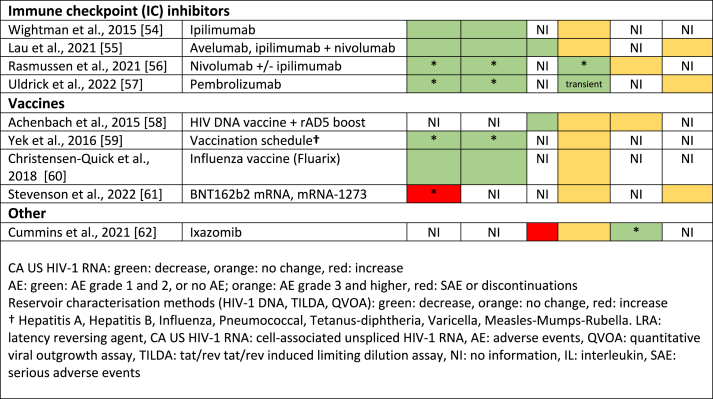
Summary of study results regarding HIV-1 reactivation and the viral reservoir of published clinical studies on people with HIV-1 exposed to latency reversing agents grouped according to their mechanism of action.

Regarding changes observed in levels of CA US HIV-1 RNA after the first LRA dose, this endpoint was reported in 48.8% of all studies and measured at a median of 24 h after treatment initiation (IQR 3–24) (Table S2). Most studies reported an increase at the first measurement after the first LRA dose, including 8 of the 10 HDACi studies and 10 of the 12 non-HDACi studies where this endpoint was reported. The four studies without such effect used vorinostat, chidamide, vesatolimod and bryostatin. 25, 37, 46, 50 Seven studies (panobinostat,^30^ romidepsin^33^, pyrimethamine,^39^ valproic acid,^39^ vorinostat,^42^ pembrolizumab,^57^ nivolumab and ipilimumab^55^^,^^56^) quantified the increase within 24 h after the first dose and reported this result separately, instead of as a pooled analysis together with changes observed after later administrations following the first dose. A significant, over 2-fold change from baseline, within this 24 h window after the first dose, was reported for pyrimethamine^39^ (2.1 after 6 h), romidepsin ^33^ (>2.5 after 4 h) and panobinostat (2.4 after 2 h).^30^ A significant effect below a 2-fold change was observed in nivolumab and ipilimumab.^56^ The subsequent dosing regimens and sampling timepoints to assess transcription reactivation following the first dose varied considerably (amongst other factors) between trials on the included LRA classes and between the drugs within a class (Table S2). This lack of uniformity precluded further comparisons of reactivation at additional timepoints.

### 3.3 Tolerability

Adverse events and/or discontinuations were registered in 38 studies (Table 3) on 666 participants that received an LRA. The use of validated scales for AE categorisation was reported in eighteen studies of which ten used the Common Terminology Criteria for Adverse Events scale,^23^^,^^27^^,^^30^, ^31^, ^32^^,^^37^^,^^39^^,^^43^^,^^44^^,^^57^ six the Division of AIDS Table for Grading the Severity of Adult and Paediatric Adverse Events,^24^^,^^25^^,^^41^^,^^52^^,^^53^^,^^58^ one a combination of the two previous scales,^35^ and one the Medical Dictionary for Regulatory Activities.^46^ Three studies reported no discontinuations or AEs: one maraviroc study with eleven participants,^47^ one vorinostat study with 16 participants,^27^ and one immune checkpoint inhibitor (ICI) study with three participants.^55^ Seven studies, on a cumulative 178 participants receiving an LRA, had participants that had to discontinue LRAs due to AEs (15.6%), including two studies on ILs (n = 72),^51^^,^^53^ and three studies on chromatin modulators (n = 87).^20^^,^^27^^,^^39^ One study investigating disulfiram and vorinostat was prematurely stopped after enrolling two participants.^42^ Eleven studies reported any AE that were grade 3 or higher. Gastro-intestinal tract AEs, fatigue, headache, and neutropenia were the most common AEs. Proportionally, at least one discontinuation occurred most frequently in the IL group (2/3 studies), and either SAEs or AEs grade 3 or higher were reported in each of the IL studies.^51^, ^52^, ^53^

**Table 3:** Reported adverse events related to the investigated LRAs according to published studies.

Author, publication year	LRA which AEs are related to	Discontinuation rate/participants receiving LRAs (%)	Total number of events		SAEs	Reported grading of AEs
			> grade 2	≤ grade 2		
Chromatin modulators						
*Histone deacetylase inhibitors (HDACis)*						
**Archin et al., 2008****^18^**	Valproic acid	0/12 (0%)	0	4	NI	NI
**Archin et al., 2010****^19^**	Valproic acid	0/8 (0%)	0	NI	NI	NI
**Routy et al., 2012****^20^**	Valproic acid	5/56 (9%)	NI	NI	NI	5 AEs-withdrawal on valproic acid (9%): mood change, GI symptoms, pulmonary emboli in 1 (2%). Only side-effect participants who withdrew described
**Archin et al., 2012****^21^**	Vorinostat	0/8 (0%)	0	NI	NI	NI
**Archin et al., 2014****^22^**	Vorinostat	0/5 (0%)	0	1	0	Mild GI symptoms; headache: no grade I severity; 5 (100%) transient platelet decline (thrombocytopenia grade 1 in 1)
**Elliott et al., 2014****^23^**	Vorinostat	0/20 (0%)	0	69	NI	51 clinical AEs (74%): 27 GI symptoms (53%) of which 8 diarrhoea (30%), 11 neurological (22%) of which 4 headache (36%) and 4 impaired concentration (36%); 18 lab AEs (26%): most frequent: 8 thrombocytopenia (44%)
**Archin et al., 2017****^24^**	Vorinostat	0/16 (0%)	0	0	0	Mild GI symptoms not grade 1; no grade I severity; 3 (13 doses, 100%) platelet decline of -15-35%
**Fidler et al., 2020****^25^**	Vorinostat	0/30 (0%)	0	28	1	1 SAE: vasovagal syncope probably related to blood draw venepuncture; Most frequent AEs: 7 diarrhoea (16%), 4 nausea (9%), 9 fatigue (21%)
**Gay et al., 2020****^26^**	Vorinostat	0/5 (0%)	0	NI	0	Mild, transient GI symptoms, not greater than grade 1
**Kroon et al., 2020****^27^**	Vorinostat	1/10 (10%)	2	115	2	34 lab AEs: most frequent: 8 decreased eGFR (24%) and 15 thrombocytopenia (44%) 81 clinical AEs:, most frequent: 9 diarrhoea (11%), 13 nausea (16%), 9 dizziness (11%) and 7 upper respiratory tract infection (9%) SAEs: treatment discontinuation in 1 (10%), hospitalisation in 2 (20%)
**Gay et al., 2022****^28^**	Vorinostat	0/8 (0%)	0	3	NI	1 nausea (33.3%), 1 diarrhoea (33.3%), 1 pruritus (33.3%)
**Scully et al., 2022****^29^**	Vorinostat	0/31 (0%)	0	2	0	1 mild dysgeusia (50%) and 1 moderate thirst (50%)
**Rasmussen et al., 2014****^30^**	Panobinostat	0/15 (0%)	0	16	0	Most frequent AEs: 8 fatigue (50%), 2 diarrhoea (13%)
**Sogaard et al., 2015****^31^**	Romidepsin	0/5 (0%)	0	35	0	Most frequent AEs: 11 nausea (31%), 4 borborygmia (11%), 2 abdominal pain (6%) and 5 fatigue (14%)
**Leth et al., 2016****^32^**	Romidepsin	0/20 (0%)	0	57	0	Most frequent AEs: 18 fatigue (32%), 24 nausea (42%), 3 vomiting (5%), 3 constipation (5%), 2 headache (4%)
**Mothe et al., 2020****^33^**	Romidepsin	0/15 (0%)	2	202	1	2 grade 4 AEs: 1 *shigella sonnei* sepsis 4 h after RMD (SAE) (0.5%), 1 creatinine kinase elevation (0.5%) Most frequent lab AEs: 8 hypophosphatemia (4%) and 5 thrombocytopenia (2%) Most frequent clinical AEs: headache/fatigue in 14 (93%), GI symptoms: nausea in 11 (73%), anorexia in 9 (60%), abdominal pain in 7 (47%), constipation/metallic taste in 6 (40%), abdominal distension in 5 (33%), vomiting in 4 (27%)
**McMahon et al., 2021****^34^**	Romidepsin	0/49 (0%)	1	24	NI	Most frequent AEs: 2 headache (16%) (grade 1), 3 fatigue (25%) (grade 2), 4 nausea (31%) (grade 2), 6 neutropenia (2 grade 1 (15%), 3 grade 2 (23%), 1 grade 3 (7%))
**Gruell et al., 2022****^35^**	Romidepsin	0/20 (0%)	1	177	1	Most frequent AEs: 38 nausea (21%), 23 headache (13%), 16 fatigue (9%), 10 chills (6%), 10 vomiting (6%), 3 prolonged QTc (2%), 7 increased QTc >10 ms post-infusion vs pre- infusion (35%) SAE: (= grade 3) increased direct bilirubin
**Gunst et al., 2022****^36^**	Romidepsin	0/28 (0%)	0	85	0	Most frequent AEs: nausea (63%) and fatigue (52%)
**Li et al., 2020****^37^**	Chidamide	0/7 (0%)	0	6	0	3 fatigue (50%), 1 rash (17%), 2 somnolence (29%)
*IKAROS Family Zinc Finger 1 protein (IKZF1) degradation*						
**Liu et al., 2022****^38^**	Lenalidomide	0/13 (0%)	0	2	0	2 (15.3%) participants developed a mild rash
*BRG-1-associated factors complex inhibitors (BAFi's)*						
**Prins et al., 2023****^39^**	Pyrimethamine, valproic acid	5/21 (%)	3	88	0	Most frequent AEs: nausea, vomiting and headache, 3 in the combination arm discontinued, 1 in pyrimethamine arm discontinued and 1 in valproic acid arm discontinued.
Transcription activation						
*Phosphatase and tensin homolog (PTEN) dysregulation*						
**Spivak et al., 2013****^40^**	Disulfiram	NI	0	NI	NI	NI
**Elliot et al., 2015****^41^**	Disulfiram	0/30 (0%)	0	76	0	62 clinical AEs (82%): 23 GI symptoms (37%) (most frequent: 6 abdominal pain (26%) and 6 diarrhoea (26%)), 16 neurological (26%) (most frequent: 6 headache (38%), 23 constitutional (37%) of which most frequent 4 sleepiness (13%), 4 fatigue (13%), 3 light-headedness (9%)); 14 lab AEs (18%): most frequent: 7 hypophosphatemia (50%)
**McMahon et al., 2022****^42^**	Disulfiram + vorinostat	2/2 (100%)	2	0	2	Grade 3 neurotoxicity in 2 leading to suspension of enrolment (SAEs): Possibly disulfiram: 1 (50%) left sigmoid sinus thrombosis, transient; Probably disulfiram/possibly VOR: 1 (50%) lethargy; dysgeusia; emotionally labile; paranoid ideation; ataxia
*Toll-like receptor (TLR) agonists*						
**Vibholm et al., 2017****^43^**	MGN1703	0/15 (0%)	1	56	0	1 grade 3 neutropenia AE; Most frequent AEs: 23 injection site reaction (40%), 7 fatigue (12%), 11 neutropenia (19%) (3 neutropenia grade 2)
**Vibholm et al., 2019****^44^**	MGN1703	NI	2	25	0	Grade 3 neutropenia in 2 participants (16%); Most frequent AEs: Grade 1 injection site reaction in 7 participants (58%), neutropenia in 9 participants (75%), grade 2 dizziness in 1 participant (8%)
**Saxena et al., 2019****^45^**	Poly-ICLC	0/12 (0%)	1	34	0	Most frequent AEs: 15 (43%) injection site reactions (10 pain (66%) and 5 erythema (33%)), 4 fever (11%) and 10 (29%) fatigue
**Riddler et al., 2020****^46^**	Vesatolimod	0/36 (0%)	0	56	0	Grade 3 neutropenia in 1 participant (3%); Most frequent AEs: fatigue in 7 (30%), nausea in 5 (22%), myalgia/headache/nasal congestion in 4 (18%)
*Non-canonical NFKb agonists*						
**Lafeuillade et al., 2014****^47^**	Maraviroc	0/11 (0%)	0	0	0	0
*Protein kinase C (PKC) agonists*						
**Gutiérrez et al., 2016****^50^**	Bryostatin	0/8 (0%)	0	5	NI	2 transient headache (20 μg arm) (40%), 2 transient myalgia (20 μg arm) (40%), 1 rash post infusion (20%) (all grade 1)
Interleukins and interleukin (IL) agonists						
**Stellbrink et al., 2002****^51^**	IL-2	1/56 (2%)	6	NI	6	AEs leading to dose reduction in 8 (31%) participants, discontinuation in 1 (4%)
**Katlama et al., 2016****^52^**	IL-7 + maraviroc	0/15 (0%)	NI	NI	1	19 ≥ grade 2 AEs in 9 participants (60% of IL-7 arm); SAE: phlebitis lower limb (right) probably related to IL-7, transient
**Miller et al., 2022****^53^**	N-803	1/16 (6%)	38	263	NI	Most frequent clinical AEs: 154 injection site reactions (51%) (34 grade 3 (22%), in 1 (6%) participant leading to withdrawal), 21 (7%) pain, 12 (4%) QTc prolongation; Most frequent lab AEs: 34 grade 1 (72%); 9 grade 2 (19%): 6 decreased eGFR (66%), 1 low Hb (11%); 4 grade 3 (9%): 2 decreased eGFR (50%) and 2 increased bilirubin (50%)
Immune checkpoint (IC) inhibitors						
**Wightman et al., 2015****^54^**	Ipilimumab	0/1 (0%)	NI	NI	NI	NI
**Lau et al., 2021****^55^**	Avelumab, ipilimumab + nivolumab	0/3 (0%)	0	0	0	0
Vaccines						
**Achenbach et al., 2015****^58^**	HIV DNA vaccine + rAD5 boost	0/14 (0%)	NI	NI	0	No severe post-vaccine reactions, only mild/moderate localised reactions: tenderness-redness-swelling at injection site; general symptoms of fatigue, malaise, and myalgia were noted after 10 (24%) DNA prime injections and 3 (21%) rAd5 boost injections
Other						
**Cummins et al., 2021****^62^**	Ixazomib	1/17 (6%)	0	5	NI	3 diarrhoea (60%), 2 maculo-papular rash (40%) with treatment interruption at 20 weeks due to maculo-papular rash in 1 participant (4 mg arm)

†Hepatitis A, Hepatitis B, Influenza, Pneumococcal, Tetanus-diphtheria, Varicella, Measles-Mumps-Rubella. LRA: Latency- reversing agent, SAE: severe adverse event, AE: adverse event, NI: no information, RMD: romidepsin, GI: gastro-intestinal, eGFR: estimated glomerular filtration rate, VOR: vorinostat, IL: interleukin, Hb: haemoglobin

### 3.4 Reservoir size and analytical treatment interruption (ATI)

The reservoir size was assessed in 42 studies. The assays used to measure the reservoir and timepoints assessed varied between studies (Tables S3–S5), but most quantified integrated or total HIV-1 DNA (90.5%). Of these, only three studies on nivolumab/ipilimumab, chidamide, and romidepsin measured a significant decline in HIV-1 DNA^32^^,^^37^^,^^56^ (Table S3). An HIV-1 DNA decline was observed with pembrolizumab and panobinostat, however it returned to baseline levels by the end of the study.^30^^,^^57^ A QVOA was performed in sixteen studies, fourteen studies reported no significant change, but a significant decrease was observed with romidepsin (−38.0%)^32^ and ixazomib (−0.94 IUPM)^62^ (Table S4). The six studies that evaluated TILDA reported no significant decreases (Table S5).^23^^,^^31^^,^^39^^,^^55^^,^^57^^,^^61^

Nine studies included an ATI in a total of 86 participants that received an LRA. There was significant heterogeneity in the criteria to restart ART and for viral rebound (Table S6). The reported median time to viral rebound ranged from 13 to 28 days, and the duration of ATI was 12–32 weeks, although some studies did not predefine a set time period. The two studies that compared an LRA to a control group did not find significant differences in time to viral rebound between groups.^27^^,^^44^ Two studies on romidepsin combined with 3BNC117 did report a significant difference in time to rebound between intervention groups.^35^^,^^36^

### 3.5 Risk of bias

Risk of bias level was high in the majority of RCTs and NRSIs. In RCTs, the risk of bias mainly arose from the randomisation process, protocol adherence and selection of reported results, while bias in NRSIs originated primarily from confounding bias and selection of reported results. (Figs. S1–S4).

## 4 Discussion

In this systematic review, we have provided an overview of the available clinical data regarding reservoir reactivation activity by CA US HIV-RNA and tolerability of different LRAs. Although study designs were heterogeneous, overall most studies confirmed that LRAs were able to reverse latency, with HDACis being the main studied drug class. Nevertheless, adverse events, including those leading to drug discontinuations, occurred across the LRA classes. Finally, we did not observe a consistent signal where one particular LRA relevantly decreased the reservoir size.

The study heterogeneity prevented firm conclusions on the most promising LRAs with regard to reactivation effect for future studies. However, we have identified some relevant signals regarding the investigated LRAs. LRAs usually exhibited their transcriptional enhancing effect within 24 h, making early sampling timepoints in a trial reasonable, also for comparison purposes between studies. The data did not provide clear guidance on when, after administration of the LRA, the maximal fold change in reactivation can be expected. The overall effect size of LRAs was an approximately two-fold increased CA US HIV-1 RNA from baseline which is modest given that the data also indicated that this was generally insufficient to induce a robust reservoir decay. As HDACis are the most frequently studied compounds, this class might prove to be useful as a reference standard, and a minimum two-fold reactivation can serve as a realistic target to aim for in future trials to identify LRAs.

The safety and tolerability of LRAs is also important for future studies. Almost 20% of the LRA trials reported at least one treatment discontinuation, with two out of the studies on ILs having discontinuations.^51^^,^^53^ Additionally, two trials reported a discontinuation rate over 10%, namely pyrimethamine with valproic acid, and disulfiram with vorinostat.^39^^,^^42^ When looking more specifically at LRA-related SAEs and high grade AEs, clustering occurred with ILs,^51^, ^52^, ^53^ as well as with some combinations of LRAs.^39^^,^^42^ This signals that pharmacovigilance remains of importance, especially when the field will advance towards combining interventional drugs in cure strategies.

Next to the ability to reactivate the reservoir, the impact on the size of the viral reservoir is of interest when evaluating LRAs. Our secondary outcomes included reservoir size, and a significant, but likely clinically irrelevant, decrease was only observed in a very limited number of studies involving nivolumab/ipilimumab, chidamide, romidepsin, and ixazomib.^32^^,^^37^^,^^56^^,^^62^ Only one study found a reservoir decrease which was confirmed by more than one reservoir quantitation assay.^32^ Interestingly, this study combined an HDACi with a vaccine (vac-4x and rhuGM-CSF), supporting the design of clinical studies using combinations of compounds that reactivate the virus and target the host immune responses.^63^

Looking at our study limitations, in line with prior reviews on the demographic features of cure trials, we have found a considerable underrepresentation of women and people with non-B HIV-1 subtypes.^64^ Only 13.3% of studies reported HIV-1 subtypes, and less than 10% of participants were women, indicating that this has not changed considerably since previous reports.^65^ This has prevented meaningful subgroup analyses. Moreover, we have found that the available baseline characteristics that were reported were often limited, study designs were heterogeneous, and confounding was frequently not considered. Other sources of potential bias resulted from the randomisation process and selection in the reporting of results in RCTs, unlike the advice given by the CONSORT 2010 statement.^66^ Confounding was an issue in most of the non-randomised studies and generally not adjusted for. Although the primers present in the assays used for the main outcomes targeted conserved viral regions, inter-laboratory variability was unknown, while assay sensitivities are known to vary. Together, this resulted in a significant bias in most studies regardless of design, which hindered interpreting results or directly comparing LRAs. Ensuring more uniformity in study designs that include HIV-1 cure interventions would help in deciding which LRA is most potent. The clinical HIV-1 field has benefited before, and is still benefiting, from uniformity in virological efficacy endpoints in antiretroviral drug registration studies. Based on these insights, an effort in future LRA studies should be made to promote diversity in the inclusion of participants, promote inter-study comparability, and tackle potential biases.

Following these observations and to help advance the field, apart from the necessity to ensure good representation in trial populations, we propose the following recommendations for future trials with the objective to find the most effective and safe LRAs. The inclusion of one or more sampling timepoints within 24 h after administration, and reporting of results, is beneficial for the uniformity in the field and at the same time minimizes the risk of bias due to drug discontinuation. Second, ensuring access to all relevant data is required for meta-analyses purposes. Third, adverse event monitoring, especially relevant when combining interventional drugs, should be standardised best by using validated scoring systems instead of relying on the investigator's interpretation only. Without the availability of an accepted standard reservoir measurement or correlate to predict post-treatment control, sufficient amount of sampling at the main timepoints provides the advantage and opportunity to perform multiple assays, store material for future use, and pool samples from different trials for exploratory analyses.

In conclusion, this systematic review confirmed the availability of a generally safe and clinically useful armamentarium of LRAs with a modest effect on HIV-1 reactivation from the latent reservoir. The need for standardised pharmacovigilance was however also apparent as was the standardisation of reporting outcomes. This will ensure more informative future clinical studies on LRAs.

## Funding

This work was supported by the Aidsfonds (grant number P-53601) and Erasmus University Medical Center (grant number FB398709 and FB393066). These funding sources had no involvement in study design, collection, analysis and interpretation of data, writing of the report or the decision to submit the article for publication.

## CRediT authorship contribution statement

**Quinten Debrabander:** Conceptualization, Data curation, Formal analysis, Investigation, Methodology, Project administration, Visualization, Writing – Original draft preparation and Editing. **Kathryn S. Hensley:** Conceptualization, Data curation, Formal analysis, Investigation, Methodology, Project administration, Visualization, Writing – Original draft preparation and Editing. **Christina K. Psomas:** Conceptualization, Writing – review & editing. **Wichor Bramer:** Methodology, Resources, Software, Validation. **Tokameh Mahmoudi:** Writing – review & editing. **Berend J. van Welzen:** Conceptualization, Visualization, Writing – review & editing, Supervision. **Annelies Verbon:** Funding acquisition, Conceptualization, Writing – review & editing, Supervision. **Casper Rokx:** Funding acquisition, Conceptualization, Project administration, Visualization, Writing – review & editing, Supervision.

## Declaration of competing interest

The authors declare the following financial interests/personal relationships which may be considered as potential competing interests: KSH received travel and conference compensation from Gilead. CR received unrestricted grants for investigator initiated research, advisory board participants, and travel compensation from ViiV and Gilead. All other authors declare no potential competing interests.

## Data Availability

Data will be made available on request.
