# Supplementary material for: The efficacy and tolerability of latency-reversing agents in reactivating the HIV-1 reservoir in clinical studies: a systematic review
Source: J Virus Erad. 2023 Aug 19;9(3):100342. doi: 10.1016/j.jve.2023.100342 (PMC10474473; doi:10.1016/j.jve.2023.100342)
Supplement: Multimedia component 1 [file mmc1.docx]

# Supplementary data

| Author, publication year, *(NCT)* | Study drugs | Male sex  n (%) | Race/ Ethnicity | Age  Median (IQR) | Acute HIV n (%)/ Fiebig Stage | HIV-1 Subtype | Nadir or pre-ART CD4+ T cell count (cells/µL)  Median (IQR) | HIV RNA zenith or pre-ART (copies/mL)  Median (IQR) | Baseline CD4+ T cell count (cells/µL) Median (IQR) | INSTI based ART regimen  n (%) | Duration of ART  Median (IQR) | Duration viral suppression  Median (IQR) |
| --- | --- | --- | --- | --- | --- | --- | --- | --- | --- | --- | --- | --- |
| Chromatin modulators | | | | | | | | | | | | |
| *Histone deacetylase inhibitors (HDACis)* | | | | | | | | | | | | |
| Archin et al., 2008 | Valproic acid | 12 (100%) | NI | NI | NI | NI | NI | NI | Range 581-1121 | NI | NI | >6m |
| Archin et al., 2010 | Valproic acid | 12 (100%) | NI | NI | NI | NI | NI | NI | >300 | NI | NI | >6m |
| Routy et al., 2012  *(NCT00289952)* | Valproic acid | 47 (84%) | NI | 4.6~ (range 28.5-80.7) | NI | NI | 218.5~ (range 17-726) | NI | 588~ (range 240-1400) | NI | 7.35y~ (range 1.2-16.1) | 51m~ (range 13-108) |
| Archin et al., 2012  *(NCT01319383)* | Vorinostat | NI | NI | NI | NI | NI | NI | NI | >300 | NI | NI | >6m |
| Archin et al., 2014  *(NCT01319383)* | Vorinostat | 16 (100%) | White (100%) | 54 (47-60) | 1 (20%)/ NI | NI | 195 (125.5-254.5) | 71928 (32177-42464210) | 705 (474.5-947) | NI | 10y (4.5-20) | 3y (2.5-5.5) |
| Elliott et al., 2014  *(NCT01365065)* | Vorinostat | 19 (95%) | NI | 47.9 (43.1-52.0) | NI | B 20 (100%) | NI | NI | 721 (621-907) | 1 (5%) | NI | 5.0y (3.9-7.5) |
| Archin et al., 2017  *(NCT01319383)* | Vorinostat | 8 (100%) | Race: White (81%), Black (19%) Ethnicity: Hispanic (6%) | 49 (33-58.5) | 3 (18%)/ NI | NI | 403 (166-526) | NI | 717.5 (556.8-783.3) | 1 (6%) | NI | 4.5y (2.2-6.1) |
| Fidler et al., 2020  *(NCT02336074)* | Vorinostat + ChAdV63.HIVconsv + MVA.HIVconsv boost | 60 (100%) | White (70%), South Asian (2%), Southeast Asian (2%), Hispanic or Latino (8%), Black Caribbean or African American (3%), Black African (3%), Mixed ethnic group (10%), Other (2%) | 32 (29-40) | 60 (100%)/ NI | B 40 (67%) A 1 (2%) F 3 (5%) Recombinant 9 (15%) Not known 7 (12%) | NI | NI | 708 (568-788) | 58 (97%) | 24 weeks<>2 years | NI |
| Gay et al., 2020  *(NCT02707900)* | Vorinostat + AGS-004 | 5 (100%) | White (100%) | 49.6 (29.3-54.4) | 1 (20%)/NI | NI | 166 (119-378.5) | NI | 542 (395-691.5) | 1 (20%) | NI | 6.1y (5.35-7.45) |
| Kroon et al., 2020  *(NCT02475915)* | Vorinostat + Maraviroc + Hydroxychloroquine | 13 (87%) | NI | 27* (range 22-51) | 15 (100%)/ Fiebig III 13 (87%), Fiebig IV 2 (13%) | CRF01_AE 14 (93%)  B 1 (7%) | 464* (range 132-740) | 5.85 log10* (range 3.1-7.5) | 865.5* (range 501-1612) | 2 (13%) | 178 weeks* (range 79-295) | ≥28 weeks |
| Gay et al., 2022  *(NCT03803605)* | Vorinostat + VRC07-523LS | 8 (75%) | Caucasian (37.5%), Black (3.5%), Hispanic (25%) | 49.5 (41.3-56.8) | NI | NI | 399 (70-568) | NI | 675 (587.5-840.5) | 7 (87.5%) | NI | 7.1y (5.2-8.5) |
| Scully et al., 2022  *(NCT03382834)* | Tamoxifen +/- vorinostat | 0 (0%) | Race: White (39%), Black (58%), American Indian or Alaskan Native (3%) Ethnicity: Hispanic (19%), Not Hispanic/Latino (81%) | 57 (53-60) | NI | NI | 232 (46-363) | NI | 688 (536-854) | 27 (87%) | 7.5y (2.9-13.9) | >1 year |
| Rasmussen et al., 2014  *(NCT01680094)* | Panobinostat | 15 (100%) | White (100%) | 47 (39-20) | NI | NI | 350 (210-410) | NI | 935 (750-1515) | 2 (13%) | 43.4m (37.6-114.8) | 38m (32.4-86.0) |
| Sogaard et al., 2015  *(NTC02092116)* | Romidepsin | 5 (83%) | Caucasian (100%) | 56 (range 36-60) | NI | NI | 250 (range 40-340) | NI | 645 (range 510-1000) | 1 (17%) | 10.1y (range 4.1-14.5) | 9.1y (range 3.6-12.7) |
| Leth et al., 2016 *(NCT02092116)* | Romidepsin + Vacc-4x + rhuGM-CSF | 17 (85%) | Caucasian (85%), African (5%), Hispanic (5%), Asian (5%) | 49 (range 32-63) | NI | NI | 280 (range 60-710) | 4.83 log10 (range 3.57-7.00) | 730 (range 593-898) | 2 (10%) | 75m (range 28-217) | 51m (range 24-183) |
| Mothe et al., 2020 *(NCT02616874)* | Romidepsin + MVA.HIVconsv vaccine | 14 (93%) | NI | 43 (range 33-51) | 14 (100%) | NI | NI | 4.9 log10 (range 3.2-5.8) | 728 (range 416-1408) | 15 (100%) | 3.23y (range 3.03-3.77) | NI |
| McMahon et al., 2021 *(NCT01933594)* | Romidepsin | 51 (91%) | White Non-Hispanic (59%), Black Non-Hispanic (36%), Hispanic (3%), Asian (2%) | 54 (range 26-72) | 0 (0%) | NI | NI | NI | 699 (range 411–1385) | 34 (58%) | NI | 6y (range 1-17) |
| Gruell et al., 2022 *(NCT02850016)* | Romidepsin +/- 3BNC117 | 17 (85%) | Caucasian (70%), African-American (25%), Hispanic (5%) | 45.5* (range 33-62) | NI | A 2 (10%)  B 14 (70%)  Unavailable 4 (20%) | NI | NI | 658* (range 450-1150) | 20 (100%) | 7.5y* (range 2-21) | >12m |
| Gunst et al, 2022 *(NCT03041012)* | Romidepsin +/- 3BNC117 | 54 (92% | Asian (8.5%), Black or African European (5.1%), White or Caucasian (81.4%), Other (5.1%) | 36 (28-27) | 28 (47.5%)/NI | B 28 (47.5)  Non-B 31 (52.5%) | 503 (range 203-1497) | 49,400 (range 730–24,000,000) | 503 (range 203-1497) | NI | NA | NA |
| Li et al., 2020 *(NCT02513901)* | Chidamide | 6 (86%) | NI | 42 (34-46) | NI | NI | NI | NI | 410 (325-473) | 0 (0%) | 36m (24-84) | 21m (18-50) |
| *IKAROS Family Zinc Finger 1 protein (IKZF1) degradation* | | | | | | | | | | | | |
| Liu et al., 2022 *(ChiCTR1900023184)* | Lenalidomide | 13 (100%) | Han Chinese (100%) | 32 (30–39) | NI | NI | NI | NI | 184 (131-342) | 10 (76.5%) | 16m (12-21) | NI |
| *BRG-1-associated factors complex inhibitors (BAFi’s)* | | | | | | | | | | | | |
| Prins et al., 2023 *(NCT03525730)* | Pyrimethamine +/-  Valproic acid | 28 (100%) | White European (86%), Latin American or Hispanic (11%), Black Caribbean (4%) | 54 (47-31) | 3 (11%)/NI | B 24 (85.7%) | 235 (160-315) | 4.9 log10 (4.7-5.3) | 665 (530-820) | 12 (43) | 7.5 y (5.6-11.7) | 6.8 (4.9-11.1) |
| Transcription activation | | | | | | | | | | | | |
| *Phosphatase and tensin homolog (PTEN) dysregulation* | | | | | | | | | | | | |
| Spivak et al., 2013 | Disulfiram | 15 (94%) | White non-Hispanic (63%), African American (31%), Hispanic (6%) | 48 (43.5-54) | NI | NI | NI | NI | 608.5 (507.5-878.5) | 0 (0%) | ≥18m | 78m (31.5-119.5) |
| Elliot et al., 2015 *(NCT01944371)* | Disulfiram | 27 (90%), (3 (10%) transgender female) | White/European-American (66.6%), Black/African-American (20%), Asian (3.3%), Hispanic/Latino (6.6%), Multi-racial (3.3%) | 54.3 (48.9-60.7) | NI | NI | NI | NI | 582 (487-728) | 5 (16.6%) | NI | >3y |
| McMahon et al., 2022 *(NCT03198559)* | Disulfiram + vorinostat | 2 (100%) | NI | 64 (61-67) | NI | NI | NI | NI | 923.5 (762-1085) | 2 (100%) | ≥3y | 11.4y (7.8-15) |
| *Toll-like receptor (TLR) agonists* | | | | | | | | | | | | |
| Vibholm et al., 2017 *(NCT02443935)* | MGN1703 | 13 (87%) | White (87%), African (13%) | 52 (46-55) | NI | NI | 212 (29-400) | NI | 583 (470-890) | 2 (13%) | 8.6y (5.25-14.08) | 3.25y (1.6-8.3) |
| Vibholm et al., 2019 *(NCT02443935)* | MGN1703 | 11 (91%) | Caucasian (91%), African (8%) | 51.5 (45-54) | 0 (0%) | NI | 330 (91-515) | NI | 635 (520-888) | 1 (8%) | 6.3y (5-9.3) | NI |
| Saxena et al., 2019 *(NCT02071095)* | Poly-ICLC | 15 (100%) | Race: White (40%), Black or African American (26.6%), Asian (6.7%), American Indian/Alaska Native(6.7%), Multiple or unspecified (20%) Ethnicity: Hispanic or Latino (46.6%), Not Hispanic or Latino (53.3%) | 39.73 (26-54) | NI | NI | NI | NI | 619 | NI | NI | ≥48 weeks |
| Riddler et al., 2020 *(NCT02858401)* | Vesatolimod | 43 (90%) | NI | 47.9* (range 23-66) | NI | NI | NI | 4.5 log 10* | 701.1* | 28 (58%) | 7.9y* | ≥12m |
| *Non-canonical NFKb agonists* | | | | | | | | | | | | |
| Lafeuillade et al., 2014 *(NCT00935480)* | Maraviroc | 17 (85%) | NI | 52~ | 0 (0%) | NI | 280~ | NI | 671~ | NI | 12m~ | 9.5m~ |
| Madrid-Elena et al., 2018 *(NCT01365065)* | Maraviroc | 19 (95%) | NI | 49 (42.5-51) | NI | NI | NI | NI | 664 (414.6-794.2) | 0 (0%) | 127.5m (58.7-181) | ≥2y |
| Lopez-Huertas et al., 2020 *(NCT00795444)* | Maraviroc | 2 (66%) | NI | 50 (35-51) | NI | NI | 230 (171-246) | NI | 746 (552-989) | 0 (0%) | 9.75y (8.5-16.83) | ≥2y |
| *Protein kinase C (PKC) agonists* | | | | | | | | | | | | |
| Gutiérrez et al., 2016 *(NCT02269605)* | Bryostatin | NI | NI | 41.5 (32-44.5) | NI | NI | 258 (181-449.5) | NI | 569.5 (487-786.5) | 1 (8%) | NI | >2y |
| Interleukins and interleukin (IL) agonists | | | | | | | | | | | | |
| Stellbrink et al., 2002 | IL-2 | 52 (93%) | Caucasian (96%), Asian (2%), African (2%) | 32* (range 18-57) | NI | NI | NI | 4.4 log10* (range 2.8-5.76) | 484* (range 186-920) | 0 (0%) | NA | NA |
| Katlama et al., 2016 *(NCT01019551)* | IL-7 +/- Maraviroc | 27 (93%) | NI | 47 (41-53) | 0 (0%) | NI | 252 (171-351) | NI | 558 (452-726) | NI | 12y (6-14) | 2.3y (2.1-2.6) |
| Miller et al., 2022 *(NCT02191098)* | N-803 | 15 (94%) | Race: White (88%), Black (6%), White/Black (6%) Ethnicity: Not Hispanic/Latino (1%), Hispanic/Latino (94%) | 48 (range 21-69) | NI | NI | NI | NI | 734 (631-1038.25) | NI | 5y (2-16.75) | >6m |
| Immune checkpoint (IC) inhibitors | | | | | | | | | | | | |
| Wightman et al, 2015 | Ipilimumab | 1 (100%) | NI | 52 (51-51) | NI | NI | 159 (159-159) | NI | 610 (610-610) | NI | 17y (17-17) | > 1 year |
| Lau et al., 2021 | Avelumab, Ipilimumab + nivolumab | 3 (100%) | NI | 68 (56-76) | NI | NI | 125 (53-210) | NI | 323 (265-468) | 1 (33%) | NI | 10y (9-11) |
| Rasmussen et al., 2021 (*NCT02408861)* | Nivolumab +/- ipilimumab | 36 (90%) | White (63%), African-American (27%) | 53 (47-58.5) | NI | NI | NI | NI | 315 (227-465) | NI | NI | NI |
| Uldrick et al., 2022 *(NCT02595866)* | Pembrolizumab | 29 (91%) | White (59%), Black (28%), Other (13%) | 55 (48.5-61.5) | NI | NI | NI | NI | 238.5 (191-425) | 22 (69%) | ≥4w | NI |
| Vaccines | | | | | | | | | | | | |
| Achenbach et al., 2015 *(NCT00976404)* | HIV DNA vaccine + rAD5 boost | 28 (100%) | White (71%) | 50 (46-55) | 0 (0%) | NI | 202 (88-280) | NI | 636 (485-790) | 0 (0%) | 13y (8-19) | 2.6y (2.2-3.0) |
| Yek et al., 2016 *(NCT00329251)* | Vaccination schedule† | 21 (81%) | NI | 39* (range 29-52) | NI | NI | 412.5* (range 373-530) | NI | 942.5* (712-1073) | NI | 3.45y* (range 1.2-6.3) | NI |
| Christensen-Quick et al., 2018 | Influenza vaccine (Fluarix) | 7 (100%) | White (86%), African-American (14%) | 59 (55-64) | 0 (0%) | NI | NI | NI | 613 (327-721) | 6 (86%) | >6m | >6m |
| Stevenson et al., 2022 | BNT162b2 mRNA, mRNA-1273 | 30 (86%) | Race: Black or African American + White (2.9%), Asian + White (2.9%), Black or African American (11.4%), White (20.0%), NA (62.9%)  Ethnicity: Arab 2.9%, Chinese 8.6%, Filipino 2.9%, Hispanic or Latinx 8.6%, Latin American 5.7%, Middle Eastern 5.7%, Not Hispanic or Latinx 28.6%, South Asian 2.9%, Southeast Asian 2.9%, White or Caucasian 31.4% | 40 (32-52) | NI | NI | 400 (165-555) | NI | 780 (574-994) | 29 (83%) | NI | NI |
| Other | | | | | | | | | | | | |
| Cummins et al., 2021 *(NCT02946047)* | Ixazomib | 16 (94%) | NI | 50.85* (range 39.3-58.2) | NI | NI | NI | NI | 783 (608-1877) | 15 (88%) | >6m | 2.6 years (1.2-4.3) |

## Table S1. Patient characteristics and demographics

*Mean of medians

~ Mean of means

¶ HBV-HAV-influenza-pneumococcus-varicella zoster virus-measles, mumps, and rubella-tetanus/diphtheria

LRA: Latency reversing agent, ART: combination antiretroviral therapy, NI: no information, NA: not applicable, m: months, y:years, w: weeks

| Author, publication year | Intervention | Dosing | Assay, Primer, Unit | Fold change CA US HIV-1 RNA after first dose | Other reported CA US HIV-1 RNA outcomes |
| --- | --- | --- | --- | --- | --- |
| Chromatin modulators | | | | | |
| *Histone deacetylase inhibitors (HDACis)* | | | | | |
| *Vorinostat* | | | | | |
| Archin et al., 2012 | Combination: NA  Control: NA | 200 mg, 400 mg  1 dose/2 weeks  Total: 3 doses (1x200mg, 2x400mg) | Assay: real-time qPCR  Primer: gag  Unit: copies/million resting CD4+ T cells | NI | Mean (range) 4.8 (1.5-10.0)** *(baseline vs 4-7h post dose 2)*  Decrease by 0.24 and 0.56 in 2 PWH ** *(4-7h post completion vs 3-4m post completion)* |
| Archin et al., 2014 | Combination: NA  Control: NA | 400 mg  3 doses/week  Total: 22 doses | Assay: real-time qPCR ddPCR (on total PBMC)  Primer: gag  Unit: qPCR: copies/million resting CD4+ T cells, ddPCR: copies/µg total PBMC-RNA | NI | qPCR: >1 (n=5)  ddPCR: median (range) 1.35 (0.62-3.02)  *(baseline vs 6h post dose 11)*  qPCR: No change (n=5)  ddPCR: median (range) 1.17 (0.46-3.26)  *(baseline vs 6h post completion)* |
| Elliott et al., 2014 | Combination: NA  Control: NA | 400 mg  1 dose/day  Total: 2 weeks | Assay: semi nested real-time qPCR  Primer: gag  Unit: copies/million 18s RNA | Mean >1*** *(baseline vs 8h post dose 1)* | Mean >1** *(baseline vs 8h, 24h, 1w, 2w, 3w, 4w, 12w post dose 1)*  Mean (95%CI) 3.00 (2.16-3.84)* *(baseline vs pooled 1w, 2w, 10w post completion)*  Mean (95%CI) 2.65 (1.76-3.52)* *(baseline vs pooled 2h, 8h, 24h, 1w, 2w post dose 1)*  >1*** *(baseline vs pooled 8h post dose 1 to 10w post completion)* |
| Archin et al., 2017 | Combination: NA  Control: NA | 400 mg  1 dose/3 days  Total: 10 doses | Assay: real-time qPCR  Primer: gag  Unit: copies/million resting CD4+ T cells | Mean >1 in 58% (7/12)*** *(baseline vs 6h post dose 1)* | Mean >1 in 83% (5/6)** *(baseline vs 6h post dose 3)*  Mean >1 in 67% (2/3)*** *(baseline vs 6h post dose 10)* |
| Fidler et al., 2020 | Combination: HIV vaccine  Control: ART | 400 mg  1 dose/3 days  Total: 28 days | Assay: semi-nested qPCR  Primer: sequence only  Unit: copies/ng RNA | Median -0.68 *(baseline vs 1d dose 1)* | Median (95%CI) difference between groups 0.02 (CI -0.19-0.24) *(baseline vs pooled 4w and 6w post completion)*  Median difference 1.5 *(baseline vs post dose 3)*  Median difference 0.06 (*baseline vs post completion)* |
| Gay et al., 2020 | Combination: dendritic cell immunotherapy  Control: NA | 400 mg  1 dose/3 days, 10 doses/cycle  Total: 2 cycles | Assay: real-time qPCR  Primer: gag  Unit: log10 copies/million resting CD4+ T cells | NI | >1 in 60% (3/5)** *(baseline vs d7-10 post completion)*  >1 in 80% *(baseline vs 4h post dose 10)* |
| Kroon et al., 2020 | Combination: maraviroc - hydroxychloroquine  Control: ART | 400 mg  1 dose/day, 2 weeks/cycle  Total: 3 cycles | Assay: semi-nested real-time qPCR  Primer: only sequence, HIV subtype B with modifications for CRF01_AE  Unit: copies/million 18s RNA | NI | >1 in 63% (5/8) in combination group *(baseline vs 2w, 6w, 10w)*  No significant difference between arms *(baseline vs 2w, 6w, 10w, time at viral rebound ATI, 24w post completion)* |
| Gay et al., 2022 | Combination: bnAb  Control: NA | 400 mg  1 dose/3 days, 10 doses/cycle  Total: 2 cycles | Assay: qPCR  Primer: gag  Unit: log10 copies/million copies TBP | NI | >1 in 38% (3/8)*** *(baseline vs post completion)* |
| Scully et al., 2022 | Combination: tamoxifen  Control: vorinostat | 400 mg  1 dose/3 days  Total: 2 doses | Assay: real-time qPCR  Primer: gag  Unit: copies/million CD4+ T cells | NI | Mean (95%) *(baseline vs 5h post completion)*  Overall: 1.2 (0.7-2.1)  TMX + VOR: 1.2 (0.6-2.3)  VOR: 1.5 (0.7-3.2) |
| *Panobinostat* | | | | | |
| Rasmussen et al., 2014 | Combination: NA  Control: NA | 20 mg  3 doses/week, every other week  Total: 8 weeks, 12 doses | Assay: semi-nested real-time qPCR  Primer: gag  Unit: copies/million unfractionated CD4+ T cells | Mean (95%CI) 2.4 (1.8-3.3)****(baseline vs 2h post dose 1)*  >1* *(baseline vs 2h and 12h post dose 1)* | >1 during treatment*** *(baseline vs pooled sampling points during treatment)*  >1 at every assayed timepoint* *(baseline vs 8h post dose, 4w and 24w post completion)* |
| *Romidepsin* | | | | | |
| Sogaard et al., 2015 | Combination: NA  Control: NA | 5 mg/m^2^  1 dose/week  Total: 3 weeks | Assay: ddPCR  Primer: gag  Unit: copies/million CD4+ T cells | >1 *(baseline vs 30min and 1d post dose 1)* | >1* *(baseline vs 30min post dose)* |
| Leth et al., 2016 | Combination:  HIV-1 vaccine + rhuGM-CSF  Control: NA | 5 mg/m^2^  1 dose/week  Total: 3 weeks | Assay: ddPCR  Primer: gag  Unit: copies/million CD4+ T cells | >1** *(baseline vs 30min post dose 1)* | >1** *(baseline vs 30min post dose)*  Mean 3.1 *(baseline vs 30min post completion)* |
| Mothe et al., 2020 | Combination: HIV-1 vaccine  Control: NA | 5 mg/m^2^  1 dose/week  Total: 3 weeks | Assay: ddPCR  Primer: gag  Unit: copies/million CD4+ T cells | Median >2.5 *(baseline vs 4h post dose 1)* | >2 in 80% *(baseline vs 4h, 8h, 24h, 72h, 7d post dose 1, 7d post dose 3)*  Median >2.5 (*baseline vs 4h post dose)* |
| McMahon et al., 2021 | Combination: NA  Control: placebo | 0.5-2-5 mg/m^2^ single dose or 5 mg/m^2^ multi dose  Multi dose: 1 dose/2 weeks  Total: 4 cycles (multi dose) | Assay: qPCR  Primer: pol  Unit: copies/million resting CD4+ T cells | >1 in single dose 0.5mg, 2mg and multi dose 5 mg cohort *(baseline vs 24h post dose)* | No significant differences across cohorts compared to placebo *(single dose cohort: baseline vs 24h and 14d post dose, multi dose cohort: baseline vs 24h and 72h post dose)* |
| Gruell et al., 2022 | Combination: 3BNC117  Control: Romidepsin | 5 mg/m^2^  1 dose/week  Total: 2 cycles of 3 weeks, 6 doses | Assay: ddPCR  Primer: gag  Unit: copies/million CD4+ T cells | Median (range)  RMD + 3BNC117: 0.93 (0.18-3.6)  RMD: 0.93 (0.60-1.21)  Overall: 0.93 (0.18-3.6)  *(baseline vs 1d post dose 1)*  RMD + 3BNC117: 0.91 (0.29-2.02)  RMD: 1.1 (0.93-1.30)  Overall: 1.02 (0.29-2.02)  *(baseline vs 7d post dose 1)* | Mean > 1 in combination group* *(baseline vs 1d post dose 3)*  Median (IQR) 1.14 (0.71-1.95)** *(baseline vs 1d post dose)* |
| *Chidamide* | | | | | |
| Li et al., 2020 | Combination: NA  Control: NA | 10 mg  2 doses/week  Total: 4 weeks | Assay: qPCR  Primer: NI  Unit: copies/million PBMCs | Median >1 *(baseline vs 6h and 2d post dose 1)* | Median >1* *(baseline vs 6h post dose 6 and post completion, 2d post completion)* |
| *IKAROS Family Zinc Finger 1 protein (IKZF1) degradation* | | | | | |
| *Lenalidomide* | | | | | |
| Liu et al., 2022 | Combination: NA  Control : NA | 25 mg  1 dose/day, 21 doses/2 days, 28 days/ cycle  Total: 12 cycles | Assay: real-time qPCR  Primer: NI  Unit: log10 copies/million PBMC | NI | Mean 0.81 *(baseline vs post completion)*  <1* *(baseline vs pooled 4w, 8w, 12w, 24w, 36w, 48w)* |
| *BRG-1-associated factors complex inhibitor (BAFi’s)* | | | | | |
| *Pyrimethamine* | | | | | |
| Prins et al., 2023 | Combination: Valproic acid, 30 mg/kg/d, 14d  Control: pyrimethamine arm, valproic acid arm, ART arm | 200 mg, 100mg  1 dose/day  Total: 14 doses (1x200mg, 13x100mg)  Valproic acid:  30 mg/kg  1 dose/day  Total: 14 doses | Assay: real-time qPCR  Primer: sequence only  Unit: US RNA/150 ng total RNA | Median (IQR)  PYR: 2.1 (1.5-2.7)*  PYR + VPA: 1.7 (1.4-1.9)*  VPA: 1.1 (0.9-1.3)  ART: 1.0 (1.0-1.5)  Pooled PYR and PYR+VPA arms 1.8 (1.4-2.4);  Pooled VPA and ART arms 1.0 (1.0-1.4);  *(baseline vs 6h post dose 1)*  No significant difference PYR vs PYR+VPA arm  Difference* pooled PYR & PYR+VPA vs VPA & ART | Difference in absolute change between groups*** *(baseline vs during and post completion)*  Difference PYR arm vs ART arm* *(baseline vs post completion)*  Increased median fold change PYR vs ART *(baseline vs every time point)*  Median (IQR)  PYR: 1.9 (1.5-2.4)* *(baseline vs 14d post initiation)*  PYR: 1.7 (1.2-2.5) *(14d post initiation vs 42d post initiation)*  ART: 1.0 (1.0-1.5) *(baseline vs 14 post initiation)*  ART: 1.0 (0.9-1.2) *(baseline vs 42d post initiation)*  VPA: no significant difference *(baseline vs 6h, 14, 42d post initiation)* |
| Transcription activation | | | | | |
| *Phosphatase and tensin homolog (PTEN) dysregulation* | | | | | |
| *Disulfiram* | | | | | |
| Elliot et al., 2015 | Combination: NA  Control: NA | 500 mg, 1000 mg, 2000 mg  1 dose/day  Total: 3 days | Assay: semi nested real-time qPCR  Primer: gag  Unit: copies/million 18s RNA | 500 mg: >1*, 1000mg: >1*** (*baseline vs 2h dose 1)*  1000mg >1** *(baseline vs 6h dose 1)*  500mg: >1***, 1000mg: >1***, 2000mg: >1** *(baseline vs 24h post dose 1)*  Mean (95%CI)  500 mg: 1.7 (1.3-2.2)***  1000 mg: 1.9 (1.6-2.4)***  2000 mg: 1.6 (1.2-2.1)**  *(baseline vs pooled 2h, 8h and post dose 1 and 3, 24h post dose 2)* | Mean (95%CI)  500 mg: 2.1 (1.5-2.9)***  1000mg: 2.5 (1.9-3.3)***  2000mg: 2.1 (1.5-3.1)***  *(baseline vs pooled d7 and d30)* |
| McMahon et al., 2022 | Combination: Vorinostat  Control: NA | 2000 mg  1 dose/day  Total: 28 days  Vorinostat:  400 mg  1 dose/day  Total: 6 doses | Assay: semi nested real-time qPCR  Primer: gag  Unit: copies/million 18s RNA | Median range 1.35 (n=2) *(baseline vs post dose dose 8 disulfiram/post dose 1 vorinostat)* | NI |
| *Toll-like receptor (TLR) agonists* | | | | | |
| *MGN1703 (TLR9)* | | | | | |
| Vibholm et al., 2017 | Combination: NA  Control: NA | 60 mg  2 doses/week  Total: 4 weeks | Assay: ddPCR  Primer: gag  Unit: copies/million CD4+ T cells | NI | <1* *(baseline vs pooled 14d and 42d post completion)*  No change *(baseline vs 24h and 48h post dose 2, 48h post dose 3, 24h post dose completion)* |
| Vibholm et al., 2019 | Combination: NA  Control: NA | 60 mg  2 doses/week  Total: 24 weeks | Assay: NI  Primer: sequence only  Unit: copies/million CD4+ T cells | NI | Median 1.27 *(baseline vs d0 post completion)* |
| *Poly-ICLC (TLR3)* | | | | | |
| Saxena et al., 2019 | Combination: NA  Control: Placebo | 1.4 mg  1 dose/day  Total: 2 days | Assay: real-time qPCR  Primer: gag  Unit: copies/µg RNA in purified CD4+ T cells | NI | No significant increase *(baseline vs 0d, 2d, 6d, 4w and 16w post completion)* |
| *Vesotalimod (TLR7)* | | | | | |
| Riddler et al., 2020 | Combination: NA  Control: Placebo | 1 mg, 2 mg, 4 mg  1 dose/2 weeks  Total: 6 doses  6 mg, 8 mg  1 dose/2 weeks  Total: 10 doses  10 mg, 12 mg  1 dose/2 weeks  Total: 10 doses (3x10mg, 7x12mg | Assay: ddPCR  Primer: pol  Unit: copies/million PBMCs | No significant change in any cohort *(baseline vs 2d post dose 1)* | No significant change in any cohort *(baseline vs 2d and 30d post completion (1-8mg cohort), 30d post completion (10-12mg cohort))*  No consistent changes related to vesotalimod *(baseline vs 2d post dose 4 (1-4mg cohort) and dose 5 (6-8mg cohort))* |
| *Non-canonical NFkB agonists* | | | | | |
| *Maraviroc* | | | | | |
| Lafeuillade et al., 2014 | Combination: Raltegravir  Control: ART | 150 mg  2 doses/day  Total: 48 weeks | Assay: qPCR  Primer: NI  Units: copies/million PBMCs | NI | Similar levels in both groups  MVC: <1 *(baseline vs 0w and 24w post completion)* |
| Madrid-Elena et al., 2018 | Combination: NA  Control: NA | 150 mg, 600 mg  2 doses/day (150 mg)  1 dose/day (600 mg)  Total: 10 days | Assay: semi-nested real time qPCR  Primer: gag  Unit: copies/million resting CD4 | Increase detected  *baseline vs d1 on treatment* | >1 *(baseline vs 3d, 10d, 28d post initiation, 18d post completion)*  >1* *(baseline vs pooled 1d, 3d, 10d, 28d post initiation)* |
| Lopez-Huertas et al., 2020 | Combination: NA  Control: NA | 150 mg, 600 mg  2 doses/day (150 mg)  1 dose/day (600 mg)  Total: 48 weeks | Assay: semi-nested real time qPCR  Primer: gag  Unit: copies/million PBMC | NI | Median (range) 0.31 (0.19-0.42) in 67% (n=2) *(baseline vs 12w post completion)*  4.30 (n=1) *(baseline vs 48w post completion)*  Median (range) 0.21 (0.03-0.35) (n=3) *(12w post initiation vs 12w post completion)*  3.65 (n=1) *(12w post initiation vs 0w post completion)*  Median (range) 4.08 (range 2.04-6.11) in 67% (n=2) *(baseline vs 12w post initiation)* |
| *Protein kinase C (PKC) agonists* | | | | | |
| *Bryostatin-1* | | | | | |
| Gutiérrez et al., 2016 | Combination: NA  Control: Placebo | 10 µg/m^2^, 20 µg/m^2^  Total: 1 dose | Assay: semi-nested real time qPCR  Primer: sequence only  Unit: levels in PBMCs | No difference between groups or time points within groups *(baseline vs 15min, 30min, 1h, 2h, 4h, 8h, 12h, 24h, 48h, 72h post dose)* | NI |
| Interleukins and interleukin (IL) agonists | | | | | |
| *IL-7* | | | | | |
| Katlama et al., 2016 | Combination: raltegravir - maraviroc  Control: raltegravir - maraviroc | 20 µg/kg  3 doses/week  Total: 3 weeks | Assay: real-time qPCR  Primer: sequence only  Unit: copies/purified CD4+ T cells | NI | No significant increase within groups *(baseline vs 2w, 18w and 46w post completion)* |
| *Immune checkpoint (IC) inhibitors* | | | | | |
| *Nivolumab-Ipilimumab-Avelumab* | | | | | |
| Wightman et al., 2015 | Ipilimumab  Combination: NI  Control: NA | 3 mg/kg  1 dose/3 weeks  Total: 12 weeks | Assay: real-time PCR  Primer: NI  Unit: copies/million 18s RNA | >1 (*Baseline vs post dose 1)* | >1 *(Baseline vs post dose 2)* |
| Lau et al., 2021 | Avelumab  Nivolumab + Ipilimumab  Combination: NA  Control: NA | Avelumab:  10 mg/kg  1 dose/2 weeks  Nivolumab + Ipilimumab:  3 mg/kg + 1mg/kg  1 dose/3 weeks  3 weeks/cycle  Total: 4 cycles | Assay: semi nested real-time qPCR  Primer: gag  Unit: copies/million 18s RNA | Mean (range) 1.3 (1.08-2.57) *(baseline vs 24h post c1)* | Mean (range)  3.1 (1.45-5.46) *(baseline vs 24h post c2)*  6.8 (1.95-13.69) *(baseline vs 24h post c3)*  8.6 (1.00-16.06) *(baseline vs 24h post c4)* |
| Rasmussen et al., 2021 | Nivolumab  Nivolumab + Ipilimumab  Combination: NA  Control : NA | Nivolumab:  3 mg/kg  1 dose/2 weeks  Single dose  Nivolumab + ipilimumab:  240 mg  1 dose/2 weeks  1 mg/kg  1 dose/6 weeks  2 weeks/cycle  Total: 4 cycles | Assay: semi nested real-time qPCR  Primer: gag  Unit: copies/million 18s RNA | Median (IQR)  NIV + IPI: 1.44 (1.16-1.89)* (*Baseline vs 24h post dose 1)*  NIV + IPI vs NIV: higher change* *(Baseline vs 24h and 7d post dose 1)* | No change in entire cohort at any time point *(Baseline vs 24h and 7d post c1 and c4)*  NIV: <1 *(Baseline vs 24h-7d post first 4 cycles)*  NIV + IPI: >1 *(24h post c1 vs 24h post c4)* |
| *Pembrolizumab* | | | | | |
| Uldrick et al., 2022 | Combination: NA  Control: NA | 200 mg  1 dose/3 weeks  Total: 35 doses | Assay: semi nested real-time qPCR  Primer: gag  Unit: copies/million 18s RNA | Mean (95%CI)  1.13 (0.79-1.61)  1.32 (1.02-1.70) (p=0.03)  1.46 (0.98-2.17)  *(Baseline vs 24h, 7d and 21d post dose 1)* | No significant change *(Baseline vs 2h post dose 2)* |
| Vaccines | | | | | |
| Christensen-Quick et al., 2018 | Influenza  Combination: NA  Control: NA | Single dose | Assay: ddPCR  Primer: gag  Unit: copies/million PBMCs | Median (range) absolute increase 256 (177-924) in 43% (n=3) *(Baseline vs pooled time points 2d, 4d, 7d post dose)* | NI |
| Yek et al., 2016 | HBV-HAV-influenza-pneumococcus-VZV-MMR-tetanus/diphteria  Combination: NA  Control: Placebo | Single dose per vaccine  1 dose/m  Total: 12m | Assay: ddPCR  Primer: gag  Unit: copies/million CD4+ T cells | Median (IQR)  Influenza/HBV: 2.4 (1.3-7.4)*  Pneumococcus/HBV: 7.0 (2.4-22.7)*  Median  VZV/HAV: 1.6  VZV/HBV: 0.6  MMR: 1.1  TD: 1.3  Placebo: No significant change  *(Baseline vs 1m post dose)* | Overall average increase per vaccine 1.7 (1.4-2.3)** vs controls 0.6 (0.1-1.6), significant difference *(baseline vs pooled 1m, 12m post dose)* |
| Stevenson et al., 2022 | SARS-CoV-2 mRNA vaccine  Combination: NA  Control: NA | 1 dose/2 weeks  Total: 2 doses | Assay: ddPCR  Primer: sequence only  Unit: copies/million C CD4+ T cells | NI | 0.6* *(Baseline vs post completion)* |

## Table S2. CA US HIV-1 RNA measurements in LRA studies

CA US HIV-1 RNA measurements are expressed in fold change, unless mentioned otherwise. When no number is mentioned after ‘dose’, the sample or time points refer to every dose administrated in the study. Abbreviations: qPCR: quantitative polymerase chain reaction, ddPCR: digital droplet polymerase chain reaction, PBMCs: peripheral blood mononuclear cells, min: minutes, h: hours, d: days, w: weeks, c: cycle, NA: not applicable, NI: no information, TBP: tata box binding protein housekeeping gene, ATI: analytical treatment interruption, ART: antiretroviral therapy, TMX: tamoxifen, VOR: vorinostat, PYR: pyrimethamine, VPA: valproic acid, MVC: maraviroc, NIV: nivolumab, IPI: ipilimumab. Statistical significance: *=p<0.05, **=p<0.01, ***= p<0.001

| Author, publication year | Study arms | Assay | Time point | Values | Conclusion |
| --- | --- | --- | --- | --- | --- |
| Chromatin modulators | | | | | |
| *Histone deacetylase inhibitors (HDACis)* | | | | | |
| Archin et al., 2014 | - Vorinostat | ddPCR | Baseline, 4w (dose 11), 8w (dose 22) | Mean HIV-1 DNA, HIV copies/1 Million Cells Baseline 210.0, dose 11 138.1, dose 22 254.0 | No significant change |
| Elliott et al., 2014 | - Vorinostat | qPCR | Baseline, 2h, 8h, 24h, 7d, 14d, 21d, 28d, 84d | No change in DNA or integrated DNA HIV DNA copies/10^6 cells: 0d-14d p=0.59 | No significant change |
| Archin et al., 2017 | - Vorinostat | qPCR | Baseline, each dose | NI | No significant change |
| Fidler et al., 2020 | - Vorinostat + ChAdV63.HIVconsv + MVA.HIVconsv boost  vaccine  - Control | qPCR | Total: baseline, 8w, 12w, 16w, 18w  Integrated: baseline, 16w or 18w | Mean total HIV DNA at 16 and 18w 3.02 log10 copies HIV DNA per 10^6 CD4+ T-cells ART-only versus 3.06 log10 copies HIV DNA per 10^6 CD4+ T-cells in ART + V + V, (mean difference of 0.04 log10 copies HIV DNA per 10^6 CD4+ T-cells [95% CI −0.03 to 0.11; p=0.26]) Mean integrated HIV DNA at 16w and 18w was 2.79 (0.51) log10 copies per 10^6 CD4+ T cells for the ART-only group and 2·83 (0·45) log10 copies per 10^6 CD4+ T cells for the ART + V + V group. ART + V + V minus ART-only was 0.05 log10 copies per 10^6 CD4+ T cells [95% CI −0.15 to 0.25; p=0.60]) | No significant change |
| Gay et al., 2020 | - Vorinostat + AGS-004 | ddPCR | Baseline, c1, c2 | NI | No significant change |
| Kroon et al., 2020 | - Vorinostat + Hydroxychloroquine + Maraviroc  - Control | qPCR | baseline, biweekly | VOR arm: Total HIV DNA at trial entry: 5.5 (0.8–93.0), end of treatment: 5.9 (0.8–45.0(copies/10^6 PBMC (p ​= ​0.75) ART arm: Total HIV DNA at trial entr: 27.0 (3.0–86.0), end of treatment: 9.0 (0.8–68.0 copies/10^6 PBMC) (p ​= ​0.62) median total DNA at viral rebound (both arms combined) in 8 (80%) 34,0 c/mil PBMC (range 1,0-105,0) | No significant change |
| Gay et al., 2022 | - Vorinostat + VRC07-523LS | IPDA | Baseline, 16w (endpoint) | No significant change was seen in total HIV DNA or nonintact proviral frequencies by IPDA | No significant change |
| Scully et al., 2022 | - Tamoxifen + vorinostat  - Vorinostat | qPCR | Baseline, after last administration of VOR (day 38) | Mean fold change  TMX+VOR arm 0.00 (95%CI 0.12-0.13),  VOR arm -0.04 (95%CI -0.33-0.25) | No change |
| Rasmussen et al., 2014 | - Panobinostat | ddPCR | Baseline, 14d, 37d, 84d | Decrease in total HIV DNA from baseline to day 14 (p=0·04; figure 3A). However, by day 37, the concentration had returned to that at baseline. | Transient decrease |
| Sogaard et al., 2015 | - Romidepsin | qPCR: total HIV DNA and 2LTR cycles, ddPCR | Baseline, 7d, 14d, 21d, 84d | One participant had a 67% decline in total HIV-1 DNA from baseline to last follow-up, we observed no overall change in total HIV-1 DNA levels indicating that the frequency of CD4+ T cells harboring total HIV DNA remained stable following romidepsin administration | No significant change |
| Leth et al., 2016 | - Romidepsin + Vacc-4x + rhuGM-CSF | Total: ddPCR, integrated: qPCR | Total: baseline, before and 6w after RMD; integrated: baseline, before and 8w after | Significant mean reduction of 39.7% (95% CI -59.7 to -11.5, p=0.012) baseline vs post romidepsin treatment 6w, no significant change in integrated DNA mean reduction 19.2% (95%CI -38.6-6.3) | Significant mean reduction |
| Mothe et al., 2020 | - Romidepsin + MVA.HIVconsv vaccine | ddPCR | Baseline, 3w, 6w, 17w | Median (range) HIV-1 DNA copies/106 CD4+ T-cells: baseline 140 (17-752), 17w 120 (11-680) p=0.0599 | No significant change |
| McMahon et al., 2021 | - 1x Romidepsin 0.5 mg/m^2^ - 5.0 mg/m^2^  - 4x Romidepsin 5.0 mg/m^2^  - Placebo | qPCR | Single dose: Baseline, 24h, 14d Multi dose: Baseline, 24h post infusion 1, Infusion 2, 24h & 72h post infusion 2, Infusion 3, 24h post infusion 3, Infusion 4, 24h post infusion 4 | No significant difference for HIV-1 DNA levels were observed in single dose cohorts compared to placebo. No significant differences were found between pre- and post-infusion time points in multi-dose cohort. | No significant change |
| Gruell et al., 2022 | - Romidepsin  - Romidepsin + 3BNC117 | ddPCR (IPDA) | Baseline, day 23, day 154 (pre-ATI) | No significant changes in the concentrations of HIV-1 DNA from baseline to midway between the treatment cycles, or to the pre-ATI time point after completion of both treatment cycles in either group. Median concentrations of intact proviral HIV-1 DNA did not change significantly from baseline to before the ATI in either group; the between group difference was also not significant | No significant change |
| Gunst et al, 2022 | - Romidepsin  - 3BNC117  - Romidepsin + 3BNC117  - Control | IPDA-like duplexed ddPCR (3dPCR) | Baseline, 180d, 365d | All four groups had a significant decrease in median intact and defective proviruses from baseline to day 365. Largest median decreases in intact proviruses among the three interventional groups, but no significant differences between the groups.  ART P=0.0005, ART+3BNC117 P=0.0002, ART+ RMD P=0.0039, ART+3BNC117+RMD P=0.0001 | No significant differences between the groups |
| Li et al., 2020 | - Chidamide | qPCR | Baseline, 14d, 27d 56d, 1y | Cell-associated HIV-1 DNA showed a mean reduction of 48.9% (95% CI: 24.4–73.5%) at day 27 (P=0.018) and 37.7% (95% CI: 12.7–62.8%) at day 56 (P= 0.028)compared with baseline levels. Maintained for approximately 1 year after the last follow-up. | Significant decrease |
| *IKAROS Family Zinc Finger 1 protein (IKZF1) degradation* | | | | | |
| Liu et al., 2022 | - Lenalidomide | qPCR | Baseline, 4w, 8w, 12w, 24w, 24w, 36w, 48w | Total HIV DNA p=0.21, BL vs 4w p=0.025, BL vs 8w p=0.071, BL vs 12w p=0.009, BL vs 24w p=0.091, BL vs 36w p=0.137, BL vs 48w p=0.224 | No significant reduction at the end of treatment |
| Transcription activation | | | | | |
| *Phosphatase and tensin homolog (PTEN) dysregulation* | | | | | |
| Elliot et al., 2015 | - Disulfiram (500mg)  - Disulfiram (1000mg)  - Disulfiram (2000mg) | qPCR | 3 timepoints pre-dose, 3d, 30d | NI | No significant change |
| McMahon et al., 2022 | - Disulfiram + vorinostat | qPCR | P1: Baseline, 1d, 8d, 11d, 59d, 197 P2: Baseline, 1d, 8d, 11d, 15d, 21d, 38d, 196d | Fold change  P1: 0.7 P2: 0.6 | No significant change |
| *Toll-like receptor (TLR) agonists* | | | | | |
| Vibholm et al., 2017 | - MGN1703 | Total HIV-1 DNA: ddPCR, Integrated: *Alu-*PCR | Baseline, 14d after last dose | Baseline mean 400.51 copies/million CD4+ T cells (IQR 181–1010) | No change |
| Vibholm et al., 2019 | - MGN1703 | ddPCR | Baseline, 24w | Total HIV-1 DNA copies per 10^6 CD4+ T cells (log) Baseline median 2.96 (IQR 2.43–3.48) - week 24 median 2.99 (IQR 2.48–3.50) P=0.34 | No significant change |
| Saxena et al., 2019 | - Poly-ICLC  - Placebo | qPCR (CA DNA) , ddPCR (IPDA) | Pre-first dose (baseline), end of study | NI | No significant change in CA-DNA or IPDA |
| Riddler et al., 2020 | - Vesatolimod 1-12mg  - Placebo | qPCR | Baseline, 4w | NI | No significant change in CA proviral DNA |
| *Non-canonical NFKb agonists* | | | | | |
| Lafeuillade et al., 2014 | - Maraviroc + RAL  - Control | RT-PCR | Baseline, 2w, 4w, 12w, 24w, 48w | NI | No change over time in the arms |
| Interleukins and interleukin (IL) agonists | | | | | |
| Stellbrink et al., 2002 | - IL-2  - Control | ddPCR (IPDA) | Baseline, dose 3 (day 7) | Pre-dose 1 measure of intact proviral DNA 73.88 (s.d., 66.73) copies per million CD4+ T cells, final time point it 105.6 (s.d., 109.1), mean difference 31.74 (s.d., 47.12), P= 0.098 | No significant change |
| Katlama et al., 2016 | - IL-7 + RAL + Maraviroc  - RAL + Maraviroc | RT-PCR | Baseline, 8w, 12w, 28w, 36w, 56w | Difference W80-W0 Median (IQR) HIV-DNA log10 copies/million PBMCs RAL/MVC -0.18 ( 0.35–0.04) p=0.064 RALL/MVC+IL7 0.03 ( 0.07–0.27) p=0.331 HIV-DNA log10 copies/million CD4+ T cells RAL/MVC -0.21 ( 0.31–0.03) p= 0.096 RALL/MVC+IL7 0.07 ( 0.05–0.29) p=0.397 HIV-DNA log10 copies/million whole blood RAL/MVC 0.21 ( 0.31–0.04) p=0.177 RALL/MVC+IL7 0.22 (0.02–0.35) p=0.022 | No reduction, increased expression of HIV DNA expressed per ml of whole blood in one arm |
| Miller et al., 2022 | - N-803 (0.3mcg/kg- 6.0mcg/kg) | qPCR | Baseline, 1m, 3m, 5m, 8m, 12m | NI | No significant difference between groups |
| Immune checkpoint (IC) inhibitors | | | | | |
| Wightman et al., 2015 | - Ipilimumab | RT-PCR | C1, c2, c3, c4 | NI | No change |
| Lau et al., 2021 | - Avelumab  - Ipilimumab + nivolumab | qPCR | Baseline, 24h and 7d (after each infusion), 8w after 4th (final) infusion | 55% reduction in HIV DNA from baseline to after the fourth infusion (could be due to assay or sampling variation and may not represent a treatment effect on the HIV reservoir) | No significant change |
| Rasmussen et al., 2021 | - Nivolumab  - Nivolumab + ipilimumab | qPCR | Baseline, 24h, 7d after every third treatment cycle (1-4-7-10) | Minor decrease in HIV DNA of around 45% within 24 hours of the first dose of ICB (P = .02) (paired t-test).  Nivolumab alone (P=.005) and nivolumab plus ipilimumab (P=.001) resulted in a statistically significant decrease in HIV DNA over the duration of the first 4 cycles of ICB (generalized negative binomial regression) | Significant decrease |
| Uldrick et al., 2022 | - Pembrolizumab | qPCR | Cycle 1, 1d, 2d, 8d, 22d | Fold change in cell-associated HIV DNA (95% CI) 1d (n=31) Ref. 2d (n=31) 0.78 (0.66, 0.93) p=0.005 8d (n=31) 0.85 (0.72, 1.01) p=0.06 22d (n=26) 0.91 (0.77, 1.08) p=0.28 | Significant decrease at day 2 and 8, return to baseline by 22d |
| Vaccines | | | | | |
| Achenbach et al., 2015 | - Treatment intensification + HIV DNA vaccine + rAD5 boost  - Treatment intensification | RT-PCR | Monthly | No significant change in total HIV DNA in the peripheral blood mononuclear cells in either study group | No significant change |
| Yek et al., 2016 | - Vaccination schedule†  - Placebo | ddPCR | Baseline, before administration and monthly (to 11m) | p=0.89 | No significant changes |
| Christensen-Quick et al., 2018 | - Influenza vaccine (Fluarix) | ddPCR | Baseline, 2d, 4d, 14d, 28d | Median of 852 copies of HIV gag DNA (range: 143–5769) per 10^6^ cells | No significant change |
| Stevenson et al., 2022 | - BNT162b2 mRNA  - mRNA-1273 | ddPCR (IPDA) | Baseline, 2w after each vaccine dose | Intact HIV p=0.24, Ψ Amplicon only p=0.22, RRE Amplicon only p=0.27 | No significant change |
| Other | | | | | |
| Cummins et al., 2021 | - Ixazomib | ddPCR | Baseline, last month of treatment | No change total HIV-1 DNA ddPCR. Intact provirus level decreased numerically on ixazomib treatment in 10/14 (71%), (median difference -10.2 copies/ 106 cells, p=0.068, Wilcoxon matched-pairs signed rank test). The median estimated half-life of intact provirus for those participants treated with ixazomib was 0.6 years (95% CI 0.3, 2.5), significantly shorter than what has been previously reported in ART-suppressed patients using the same assay, performed in the same lab (7.1 years (95% CI 3.9, 18), p=0.004, one sample Wilcoxon Signed Rank Test) | No significant change in HIV DNA, non-significant reduction in IPDA |

## Table S3: DNA measurements

† Hepatitis A, Hepatitis B, Influenza, Pneumococcal, Tetanus-diphtheria, Varicella, Measles-Mumps-Rubella. LRA: Latency reversing agent, NI: no information, RMD: romidepsin, GI: gastro-intestinal, RAL: raltegravir, MVC: maraviroc VOR: vorinostat, TMX: , IL: interleukin, c: cycle, d:days, w: weeks, RRE: rev response element, Ψ: packaging signal

| Author, publication year | Study arms | n/n (%) | Time point | Values | Conclusion |
| --- | --- | --- | --- | --- | --- |
| Chromatin modulators | | | | | |
| *Histone deacetylase inhibitors (HDACis)* | | | | | |
| Archin et al., 2008 | - Valproic acid | 11/12 (92%) | Baseline (-49d,-27d), 12-16w (pooled data from two assays pre-VPA and two assays after addition of VPA) | Median change -30 (IQR -180;26) IUPB | Depletion of RCI of more than 50% in 4 participants (36%)* |
| Archin et al., 2010 | - Valproic acid  - Valproic acid + Raltegravir  - Valproic acid + Enfurvirtide  - Enfurvitide | 12/12 (100%) | Baseline, 32w | Median change 35 (IQR -35.5;77.5) IUPB | Depletion of RCI of more than 50% in 6 participants (50%) |
| Routy et al., 2012 | - Valproic acid (16 weeks)  - Valproic acid (32 weeks) | 42/56 (75%) | Baseline, 16w, 48w | 16w VPA treatment: median IUPB (log10) baseline 2.55 (range 1.20-4.20), 1.80 (range 1.0-4.70), 2.70 (range 1.0-3.90) P=0.87  32w VPA treatment: median IUPB (log10) 2.55 (range 1.20-4.65) - 1.64 (range 1.0-4.48), - 2.51 (range 1.0-4.48) p=0.50 | No significant change |
| Archin et al., 2014 | - Vorinostat | 5/5 (100%) | Baseline, 4w (after 11 doses), 8w (after 22 doses) | NI | No significant change |
| Archin et al., 2017 | - Vorinostat | 7/12 (58%) | baseline, after 2nd cycle (paired doses) and after 13 doses | NI | No significant change (defined as >0.3 log10 reduction) |
| Fidler et al., 2020 | - Vorinostat + ChAdV63.HIVconsv + MVA.HIVconsv boost  vaccine  - Control | 51/60 (85%) | Baseline, 16w | Median difference (VOR-ART arm) between groups at 16w:  With detectable QVOA: 0.05 IUPM (range -0.28-0.39) p=0.76  All participants: 0.13 log10 IUPM (range -0.38-0.65) p=0.61 | No statistically significant change |
| Gay et al., 2020 | - Vorinostat + AGS-004 | NI/5 | Baseline, after 2 cycles, mean cycle duration=28w | NI | No significant change |
| Gay et al., 2022 | - Vorinostat + VRC07-523LS | 8/8 (100%) | Baseline, 16w | No QVOA reductions exceeded the 50% threshold. | No significant change |
| Rasmussen et al., 2014 | - Panobinostat | 15/15 (100%) | Baseline, 4w after treatment | Mean 1.22 (95% 0.49-1.95) vs 1.21 (95% 0.66-1.78) (p=0.08) IUPM | No significant change |
| Sogaard et al., 2015 | - Romidepsin | 6/6 (100%) | Baseline, 6w after RMD treatment | NI | No significant change |
| Leth et al., 2016 | - Romidepsin + Vacc-4x + rhuGM-CSF | 6/20 (30%) | Baseline, 2w before RMD (after Vacc-4x and rhuGMCSF), 6w after RMD | -38.0% (95% -67.0;-8.0) (p=0.019) (Baseline - 6w after RMD treatment) | Significant decrease |
| Transcription activation | | | | | |
| *Phosphatase and tensin homolog (PTEN) dysregulation* | | | | | |
| Spivak et al., 2013 | - Disulfiram | 15/16 (94%) | -2w, +10w (before and after treatment) | Fold change 1.16 (95%CI 0.70-1.92, p=0.56) | No decrease |
| *Toll-like receptor (TLR) agonists* | | | | | |
| Vibholm et al., 2017 | - MGN1703 | 10/15 (67%) | Baseline, 14d after last treatment | NI | No change |
| Vibholm et al., 2019 | - MGN1703 | 12/12 (100%) | Baseline, 24w after treatment | 0.19 (0.08–0.26) - 0.18 (0.11–0.85) (p=0.38) | No significant change |
| Immune checkpoint (IC) inhibitors | | | | | |
| Rasmussen et al., 2021 | - Nivolumab  - Nivolumab + ipilimumab | 10/40 (25%) | Baseline, cycle 16, termination | p=0.375 | No significant decrease |
| Other | | | | | |
| Cummins et al., 2021 | - Ixazomib | 15/17 (88%) | Visit 2 (before treatment), visit 13 (near the end of study treatment) | Median difference -0.05 IUPM (p=0.48) (n=15 whole cohort)  Median difference -0.94 IUPM (p=0.031) (n=6 with baseline IUPM>1 completion of per protocol ixazomib cycles) | Significant decrease (in subgroup) |

## Table S4: QVOA

* The depletion of RCI seen at week 16 regressed to baseline at 48 and 96 weeks (follow-up in Archin, 2010)

QVOA: quantitative viral outgrowth assay, LRA: Latency reversing agent, ART: antiretroviral therapy, NI: no information, rHuGM-CSF: recombinant human granulocyte-macrophage colony-stimulating factor, RMD: romidepsin, VPA: valproic acid, VOR: vorinostat, RAL: raltegravir, IQR: interquartile range, IUPM: infectious units per million, IUPB: infectious units per billion, w: weeks, d: days, RCI: resting cell infection, BL: baseline

| Author, publication year | Study arms | TILDA performed n/n (%) | Time point | Values | Conclusion |
| --- | --- | --- | --- | --- | --- |
| Chromatin modulators | | | | | |
| *Histone deacetylase inhibitors (HDACis)* | | | | | |
| Elliott et al., 2014 | - Vorinostat | 6/20 (30% | Baseline, 1d, 14d, 84d | NI | No change |
| Sogaard et al., 2015 | - Romidepsin | Baseline 4/6 (66.7%)  6w after treatment 6/6 (100%) | Baseline, 6w after RMD treatment | n=4 with before and after samples:  2/4 participants 1–2% decrease  2/4 participants 49–83% decrease | No substantial reductions |
| *BRG-1-associated factors complex inhibitors (BAFi’s)* | | | | | |
| Prins et al., 2023 | - Pyrimethamine  - Valproic acid  - Valproic acid + Pyrimethamine  -Control | 20/28 (71%) | Baseline, 42d, ≥1y | PYR: 5.1 cells/million CD4+T- cells (range 2.7 to 109.8) before - 10.0 cells/million CD4+T- cells (range 1.6 to 122.8) after treatment  VPA: median 14.8 cells/million CD4+T-cells at day 0 and median 19.4 CD4+T-cells /million at day 42 | No significant change |
| Immune checkpoint (IC) inhibitors | | | | | |
| Lau et al., 2021 | - Avelumab  - Ipilimumab + nivolumab | 3/3 (100%) | Baseline, end of study | P1 and P2 no quantifiable level. P3 33% reduction (could be due to assay or sampling variation) | No consistent changes |
| Uldrick et al., 2022 | - Pembrolizumab | 23/32 (72%) | Baseline, EOT c7, c7, EOT ≥ c7, c13 | Fold change (95% CI)  Baseline (n=23) ref  EOT c7 (n=13) 0.77 (0.61,0.91) p=0.02  c7 (n=10) 1.40 (0.09,1.80) p=0.008  EOT ≥ c7 (n=6) 1.22 (0.76-1.95) p=0.41  c13 (n=5) 0.75 (0.55,1.03) p=0.07 | Increase in the frequency  of cells |
| Vaccines | | | | | |
| Stevenson et al., 2022 | - BNT162b2 mRNA  - mRNA-1273 | 4/35 (11%) | Baseline, 2w after vaccine dose 1, 2w after vaccine dose 2 | 1 unchanged, 1 undetectable, 2 with inducible cells trending higher across visits | No reduction in inducible cells |

## Table S5: Tat/rev induced limiting dilution assay (TILDA)

LRA: Latency reversing agent, TILDA: Tat/rev induced limiting dilution assay, RMD: romidepsin, PYR: pyrimethamine, VPA: valproic acid, h:hour, d: days, w: weeks, y: year NI: no information, EOT: end of treatment, <C7: before cycle 7, ≥C7: on or after cycle 7, C13: cycle 13

| Author, publication year | Study arms | Study participants | Participants on LRA/ number in ATI (%) | Definition of viral rebound/ restart ART | Duration of ATI | Time to viral rebound  Median (range) | Conclusion |
| --- | --- | --- | --- | --- | --- | --- | --- |
| Chromatin modulators | | | | | | | |
| *Histone deacetylase inhibitors (HDACis)* | | | | | | | |
| Kroon et al., 2020 | - Vorinostat + Hydroxychloroquine + Maraviroc  - Control | 15 | 9/14 (64%) | >1000 c/mL | 24 weeks | 28 days (14-49) | No difference between VOR arm and ART arm All participants reinitiated ART and had suppressed viremia at the end of the study |
| Rasmussen et al., 2014 | - Panobinostat | 15 | 9/9 (100%) | >1000 c/mL | NI | 17 days (17-24.5) | No correlation in time to rebound and baseline total/integrated HIV DNA. Significant correlation in time to rebound and change in total/ integrated DNA |
| Leth et al., 2016 | - Romidepsin + Vacc4x + rhuGM-CSF | 20 | 16/16 (100%) | >1000 c/mL | 16 weeks | 25 days (IQR 17-29) | NI |
| Mothe et al., 2020 | - Romidepsin + MVA.HIVconsv vaccine* | 15 | 14/14 (100%) | >20 c/mL | 32 weeks | 13 days (7-35) | 3 participants remained off ART with sustained pVL <2,000 copies/ml for a total of 32 weeks. At week 32, two out of the three stayed off cART. One showed a late rebound after 48 weeks off cART and the other one, voluntary resumed cART after 1.5 years off cART despite sustained low-level viremia. |
| Gruell et al., 2022 | - Romidepsin  - Romidepsin + 3BNC117 | 20 | 7/17 (41%) | >200 c/ml on 2 consecutive measurements | 24 weeks | BnAb/RMD: 18 days (IQR 14-42)  RMD: 28 days (IQR 21-35) | RMD only: <35 days for 16 (94%), 84 days for 1 (6%)  Significant difference in time to viral rebound between two groups (p=0.016) but not clinically relevant |
| Gunst et al., 2022 | - Romidepsin  - 3BNC117  - Romidepsin + 3BNC117  - Control | 59 | 11/20 (34%) | >5000 c/ml on 2 consecutive measurements | 12 weeks | NI | 7/20 individuals did not meet the criteria for viral rebound and displayed partial ART-free virologic control with viremia ranging from <20 to 4,060 copies per ml at week 12.  Significant difference between 3BNC117-sensitive individuals to all other ATI participants (p=0.025) |
| Transcription activation | | | | | | | |
| *Toll-like receptor (TLR) agonists* | | | | | | | |
| Vibholm et al., 2019 | - MGN1703 during ATI  - No treatment during ATI | 12 | 9/9 (100%) | >5000 c/mL | NI | 21 days | No difference in time to rebound was observed between the ATI arms (log rank P=0.25). One of nine ATI participants, despite harboring a large replication-competent reservoir, controlled viremia for 150 days |
| *Non-canonical NFKb agonists* | | | | | | | |
| Lopez-Huertas et al., 2020 | - Maraviroc | 3 | 3/3 (100%) | >200 c/mL | NI | 21 days (14-35) | NI |
| Interleukins and interleukin (IL) agonists | | | | | | | |
| Stellbrink et al., 2002 | - IL-2  - Control | 59 | 8/13 (62%) | Rebound to pre-treatment levels of HIV viral load | NI | NI | IL-2 group: 3 at 28d, 4 at 56d, 6 at 70d, 7 at 98d No IL-2: 1 at 14d, 4 at 28d, 5 at 56d Only one IL-2-treated patient rebounded to a level of plasma viraemia 0.4 log10 copies/ml lower than before therapy at the last follow-up at day 133. |

## Table S6: Analytical Treatment Interruption

LRA: Latency reversing agent, NI: no information, ART: antiretroviral therapy, ATI: Analytical treatment interruption, IL: interleukin, RMD: romidepsin, BnAb: broadly neutralising antibody


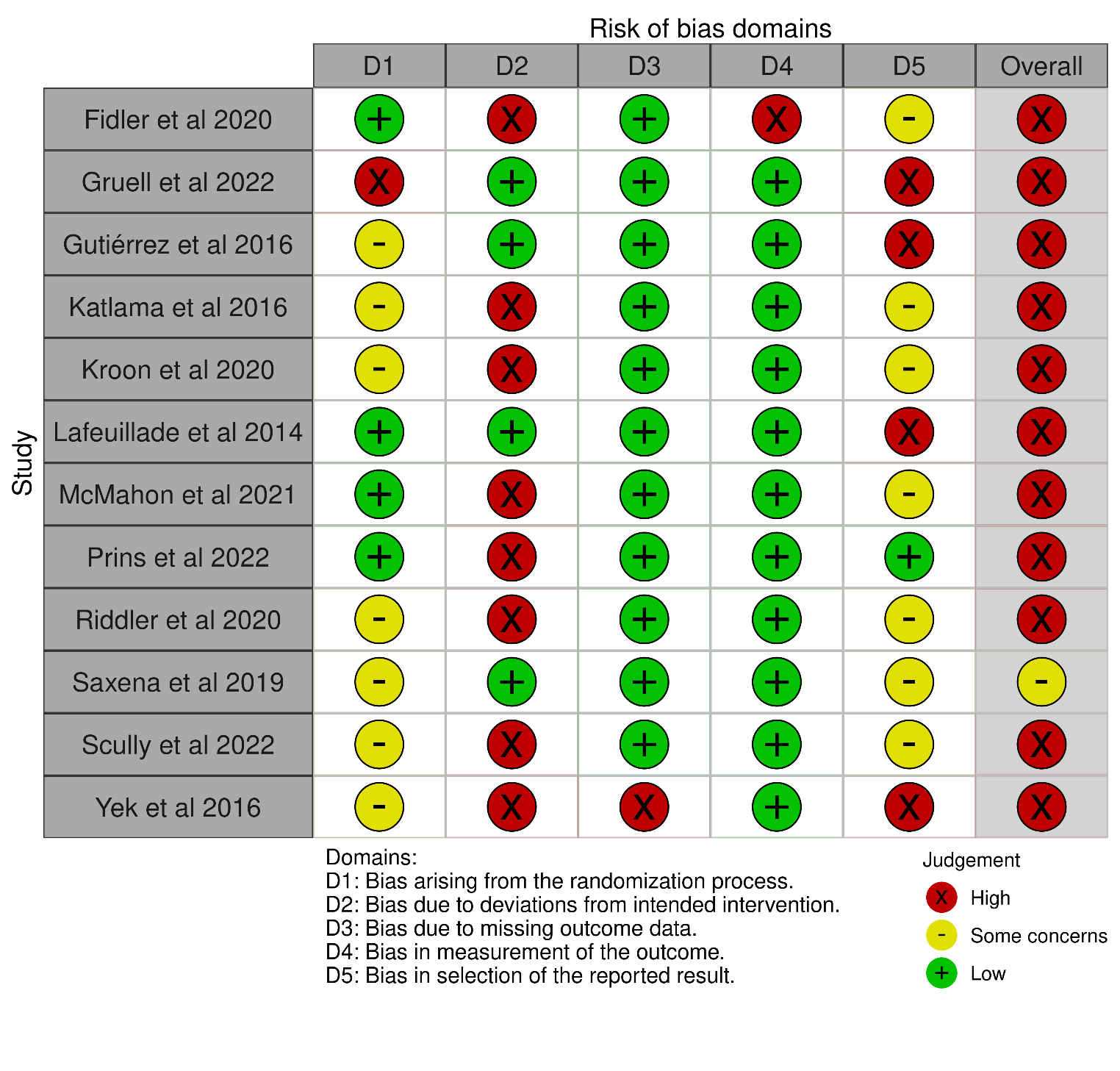


## Figure S1. Risk of bias amongst randomised studies using the Version 2 of the Cochrane risk-of-bias tool for RCTs (RoB 2)

*(2-column fitting image)*


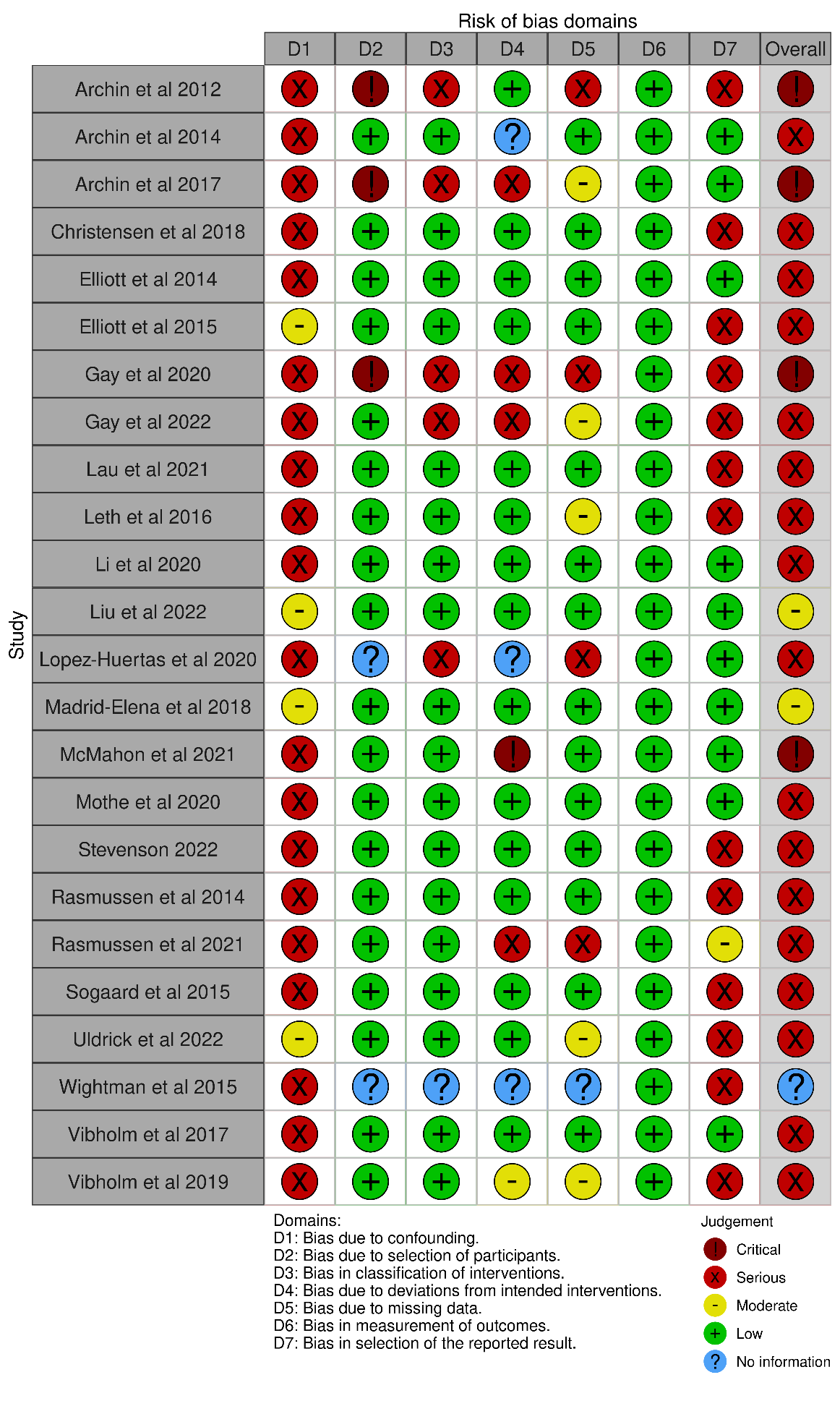


## Figure S2. Risk of bias for non-randomised studies using the Risk of Bias in Non-randomised Studies of Interventions (ROBINS-I) tool

*(2-column fitting image)*


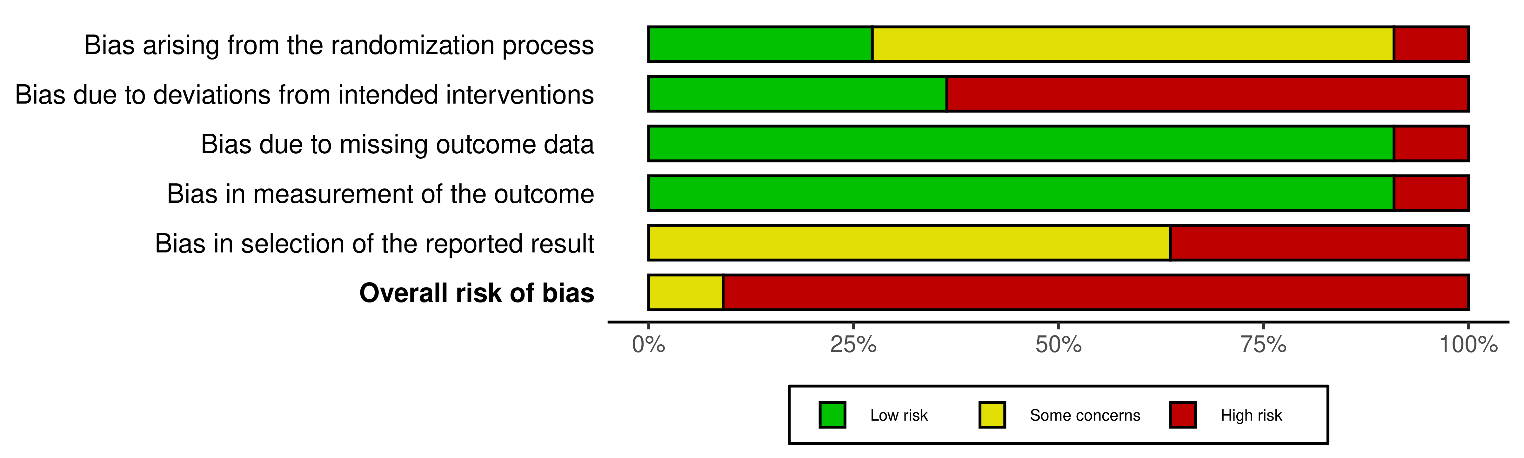


## **Figure S3. Summary plot of risk of bias amongst randomised studies using RoB2**

*(2-column fitting image)*


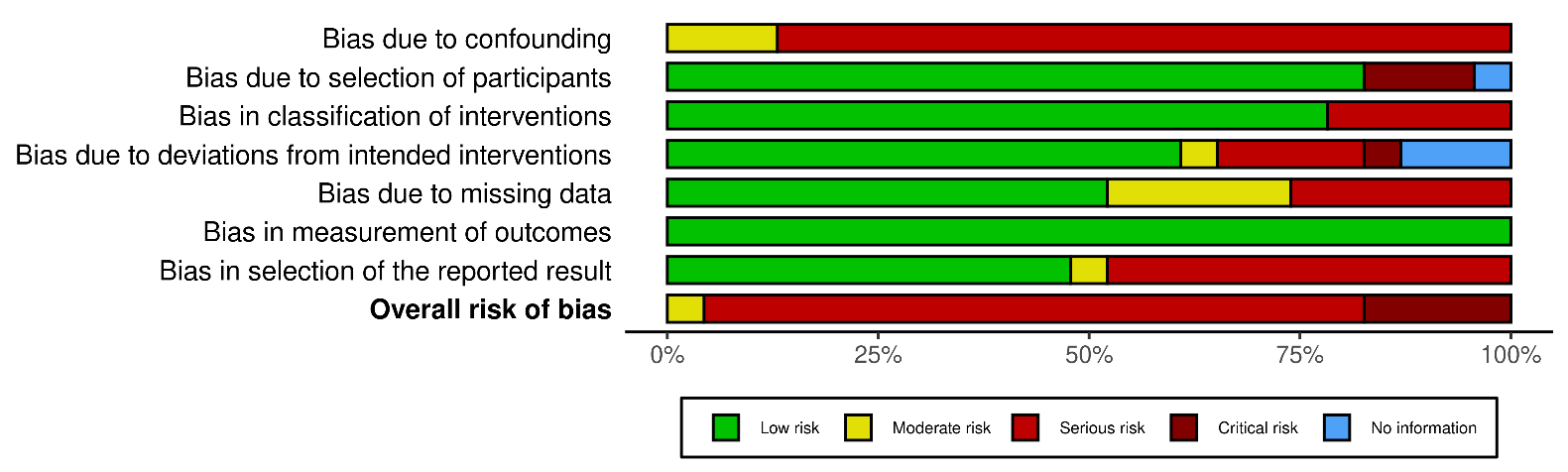


## Figure S4. Summary plot of risk of bias amongst randomised studies using ROBINS-I

*(2-column fitting image)*

## Appendix 1. PRISMA Checklist

| **Section and Topic** | **Item #** | **Checklist item** | **Location where item is reported** |
| --- | --- | --- | --- |
| **TITLE** | | |  |
| Title | 1 | Identify the report as a systematic review. | Title |
| **ABSTRACT** | | |  |
| Abstract | 2 | See the PRISMA 2020 for Abstracts checklist. | Abstract |
| **INTRODUCTION** | | |  |
| Rationale | 3 | Describe the rationale for the review in the context of existing knowledge. | Introduction, paragraph 1-3 |
| Objectives | 4 | Provide an explicit statement of the objective(s) or question(s) the review addresses. | Introduction, paragraph 4 |
| **METHODS** | | |  |
| Eligibility criteria | 5 | Specify the inclusion and exclusion criteria for the review and how studies were grouped for the syntheses. | Eligibility criteria |
| Information sources | 6 | Specify all databases, registers, websites, organisations, reference lists and other sources searched or consulted to identify studies. Specify the date when each source was last searched or consulted. | Search Strategy and Study Selection, paragraph 2 |
| Search strategy | 7 | Present the full search strategies for all databases, registers, and websites, including any filters and limits used. | Appendix 2 |
| Selection process | 8 | Specify the methods used to decide whether a study met the inclusion criteria of the review, including how many reviewers screened each record and each report retrieved, whether they worked independently, and if applicable, details of automation tools used in the process. | Search Strategy and Study Selection, paragraph 2 |
| Data collection process | 9 | Specify the methods used to collect data from reports, including how many reviewers collected data from each report, whether they worked independently, any processes for obtaining or confirming data from study investigators, and if applicable, details of automation tools used in the process. | Quality Assessment and Data Extraction, paragraph 2 |
| Data items | 10a | List and define all outcomes for which data were sought. Specify whether all results that were compatible with each outcome domain in each study were sought (e.g. for all measures, time points, analyses), and if not, the methods used to decide which results to collect. | Quality Assessment and Data Extraction, paragraph 2 |
|  | 10b | List and define all other variables for which data were sought (e.g. participant and intervention characteristics, funding sources). Describe any assumptions made about any missing or unclear information. | Quality Assessment and Data Extraction, paragraph 2 |
| Study risk of bias assessment | 11 | Specify the methods used to assess risk of bias in the included studies, including details of the tool(s) used, how many reviewers assessed each study and whether they worked independently, and if applicable, details of automation tools used in the process. | Quality Assessment and Data Extraction, paragraph 1 |
| Effect measures | 12 | Specify for each outcome the effect measure(s) (e.g. risk ratio, mean difference) used in the synthesis or presentation of results. | Endpoints and Data Synthesis |
| Synthesis methods | 13a | Describe the processes used to decide which studies were eligible for each synthesis (e.g. tabulating the study intervention characteristics and comparing against the planned groups for each synthesis (item #5)). | Endpoints and Data Synthesis |
|  | 13b | Describe any methods required to prepare the data for presentation or synthesis, such as handling of missing summary statistics, or data conversions. | Endpoints and Data Synthesis |
|  | 13c | Describe any methods used to tabulate or visually display results of individual studies and syntheses. | Quality Assessment and Data Extraction, paragraph 2 |
|  | 13d | Describe any methods used to synthesize results and provide a rationale for the choice(s). If meta-analysis was performed, describe the model(s), method(s) to identify the presence and extent of statistical heterogeneity, and software package(s) used. | Endpoints and Data Synthesis |
|  | 13e | Describe any methods used to explore possible causes of heterogeneity among study results (e.g. subgroup analysis, meta-regression). | Not reported |
|  | 13f | Describe any sensitivity analyses conducted to assess robustness of the synthesized results. | Not reported |
| Reporting bias assessment | 14 | Describe any methods used to assess risk of bias due to missing results in a synthesis (arising from reporting biases). | Not reported |
| Certainty assessment | 15 | Describe any methods used to assess certainty (or confidence) in the body of evidence for an outcome. | Not reported |
| **RESULTS** | | |  |
| Study selection | 16a | Describe the results of the search and selection process, from the number of records identified in the search to the number of studies included in the review, ideally using a flow diagram. | Figure 1 |
|  | 16b | Cite studies that might appear to meet the inclusion criteria, but which were excluded, and explain why they were excluded. | Characteristics of Included Studies, Figure 1 |
| Study characteristics | 17 | Cite each included study and present its characteristics. | Table 1 |
| Risk of bias in studies | 18 | Present assessments of risk of bias for each included study. | Risk of bias, Figures S1-4 |
| Results of individual studies | 19 | For all outcomes, present, for each study: (a) summary statistics for each group (where appropriate) and (b) an effect estimate and its precision (e.g. confidence/credible interval), ideally using structured tables or plots. | Table 3, Table S1-6 |
| Results of syntheses | 20a | For each synthesis, briefly summarise the characteristics and risk of bias among contributing studies. | Table 2, Characteristics of included studies , HIV reservoir activity, Tolerability, Reservoir size and analytical treatment interruption, Risk of bias |
|  | 20b | Present results of all statistical syntheses conducted. If meta-analysis was done, present for each the summary estimate and its precision (e.g. confidence/credible interval) and measures of statistical heterogeneity. If comparing groups, describe the direction of the effect. | Not reported |
|  | 20c | Present results of all investigations of possible causes of heterogeneity among study results. | Not reported |
|  | 20d | Present results of all sensitivity analyses conducted to assess the robustness of the synthesized results. | Not reported |
| Reporting biases | 21 | Present assessments of risk of bias due to missing results (arising from reporting biases) for each synthesis assessed. | Not reported |
| Certainty of evidence | 22 | Present assessments of certainty (or confidence) in the body of evidence for each outcome assessed. | Not reported |
| **DISCUSSION** | | |  |
| Discussion | 23a | Provide a general interpretation of the results in the context of other evidence. | Discussion, paragraph 1-4 |
|  | 23b | Discuss any limitations of the evidence included in the review. | Discussion, paragraph 5 |
|  | 23c | Discuss any limitations of the review processes used. | Discussion, paragraph 5 |
|  | 23d | Discuss implications of the results for practice, policy, and future research. | Discussion, paragraph 6-7 |
| **OTHER INFORMATION** | | |  |
| Registration and protocol | 24a | Provide registration information for the review, including register name and registration number, or state that the review was not registered. | Search Strategy and Study Selection, paragraph 1 |
|  | 24b | Indicate where the review protocol can be accessed, or state that a protocol was not prepared. | Search Strategy and Study Selection, paragraph 1 |
|  | 24c | Describe and explain any amendments to information provided at registration or in the protocol. | Amendments to the protocol can be found in the Prospero database record ID CRD42022341021. |
| Support | 25 | Describe sources of financial or non-financial support for the review, and the role of the funders or sponsors in the review. | Page 1, funding |
| Competing interests | 26 | Declare any competing interests of review authors. | Page 1, declaration of interest |
| Availability of data, code, and other materials | 27 | Report which of the following are publicly available and where they can be found: template data collection forms; data extracted from included studies; data used for all analyses; analytic code; any other materials used in the review. | Not reported |

*From:*  Page MJ, McKenzie JE, Bossuyt PM, Boutron I, Hoffmann TC, Mulrow CD, et al. The PRISMA 2020 statement: an updated guideline for reporting systematic reviews. BMJ 2021;372:n71. doi: 10.1136/bmj.n71

For more information, visit: <http://www.prisma-statement.org/>

## Appendix 2. Search information and string

| **Database searched** | **Platform** | **Years of coverage** | **Records** | **Records after duplicates removed** |
| --- | --- | --- | --- | --- |
| Medline ALL | Ovid | 1946 - Present | 2655 | 2639 |
| Embase | Embase.com | 1971 - Present | 3788 | 1405 |
| Web of Science Core Collection* | Web of Knowledge | 1975 - Present | 3146 | 782 |
| Cochrane Central Register of Controlled Trials** | Wiley | 1992 - Present | 671 | 356 |
| **Total** | | | **10260** | **5182** |

*Science Citation Index Expanded (1975-present) ; Social Sciences Citation Index (1975-present) ; Arts & Humanities Citation Index (1975-present) ; Conference Proceedings Citation Index- Science (1990-present) ; Conference Proceedings Citation Index- Social Science & Humanities (1990-present) ; Emerging Sources Citation Index (2005-present)

** Manually deleted abstracts from trial registries

No other database limits were used than those specified in the search strategies

## Embase.com

('Human immunodeficiency virus'/exp OR 'Human immunodeficiency virus infection'/exp OR 'Human immunodeficiency virus protein'/exp OR 'anti human immunodeficiency virus agent'/exp OR 'Human immunodeficiency virus infected patient'/de OR ((Human NEXT/1 (immunodeficienc* OR immun*-deficienc*) NEXT/1 virus*) OR hiv OR (acquired NEXT/1 (immunodeficienc* OR immun*-deficienc*) NEXT/1 syndrome*) OR aids):Ab,ti) AND ('latency reversing agent'/de OR (('virus latency'/de OR 'latent virus infection'/de OR 'latent period'/de) AND ('virus reactivation'/de OR 'virus activation'/de)) OR (((Laten* OR reservoir*) NEAR/3 (revers* OR reactivat* OR activat*)) OR (shock NEAR/3 kill*)):ab,ti,kw,de OR (('histone deacetylase inhibitor'/exp OR 'histone lysine methyltransferase inhibitor'/exp OR 'DNA methyltransferase inhibitor'/exp OR 'toll like receptor agonist'/exp OR 'bromodomain inhibitor'/exp OR 'interleukin 2'/de OR 'interleukin 6'/de OR 'interleukin 7'/de OR 'interleukin 21'/de OR 'tumor necrosis factor'/de OR ((Latency NEAR/3 revers*) OR (shock NEAR/3 kill*) OR ((hdac OR bromodomain* OR Bet) NEAR/3 inhibit*) OR ((histone) NEAR/3 (deacetylase OR methyltransferase OR methyl-transferase) NEAR/6 inhibit*) OR ((DNA OR desoxyribonucle*) NEAR/3 (methyltransferase OR methyl-transferase) NEAR/6 inhibitor*) OR (BRG NEAR/3 Associated-Factors NEAR/3 Inhibitor*) OR deazaneplanocin-A* OR abexinostat* OR ACSS2-agonist* OR agatolimod* OR alobresib* OR alteminostat* OR apabetalon* OR apicidin* OR AZ391 OR azacitidin* OR Azacytidine* OR Azadeoxycytidin* OR belinostat* OR birabresib* OR BIX-01294 OR bromosporin* OR butyric-acid* OR cavrotolimod* OR CG05 OR CG06 OR Chaetocin* OR Chidamid* OR citarinostat* OR cobitolimod* OR cpg-10101 OR cxd-101 OR dacinostat* OR decitabin* OR depudecin* OR entinostat* OR entolimod* OR epetirimod* OR EPZ-6438 OR fimepinostat* OR flucytosine-deoxyribosid* OR gardiquimod* OR givinostat* OR glucopyranosyl-lipid-A* OR GSK-34 OR guadecitabin* OR guretolimod* OR hydralazin* OR imiquimod* OR inobrodib* OR isatoribin* OR ivaltinostat* OR JMF1080 OR lapretolimod* OR Largazoles* OR lefitolimod* OR lirametostat* OR litenimod* OR loxoribin* OR mivebresib* OR mocetinostat* OR molibresib* OR motolimod* OR phthaloyltryptophan* OR nanatinostat* OR oxamflatin* OR panobinostat* OR pelabresib* OR pinometostat* OR pivaloyloxymethyl-butyrat* OR pracinostat* OR quisinostat* OR rec-2282 OR remetinostat* OR resiquimod* OR resiquimod-pegol* OR resminostat* OR ricolinostat* OR rintatolimod* OR roducitabin* OR romidepsin* OR SDL148 OR SDL256 OR selgantolimod* OR sirtinol* OR Sodium-crotonat* OR sotirimod* OR tacedinalin* OR tazemetostat* OR tefinostat* OR telratolimod* OR Thiophenyl-benzamid* OR tributyrin* OR trichostatin-A* OR trotabresib* OR tubacin* OR tucidinostat* OR UNC-0638 OR UNC-1999 OR UNC-926 OR valemetostat* OR verpasep-caltespen* OR vesatolimod* OR vidutolimod* OR vorinostat* OR zebularin* OR 3-ingenol* OR 3M-002 OR 7SK-snRNP-disrupter* OR 8-azaguanin* OR ABX464 OR Abyssomicin-2 OR AG555 OR AGK2 OR Alpha-CD28 OR Alpha-CD3 OR Anti-CTLA-4 OR Anti-PD1 OR Apicidin* OR Aplysiatoxin* OR As2O3 OR Baf-inhibitor* OR Belinostat* OR Benzotriazole* OR Birinapant* OR BMS-936559 OR BRD3308 OR Bryolog* OR Bucladesin* OR Cardiac-glycoside-aglycone* OR Carfilzomib* OR Cemiplimab* OR CL413 OR Clomifen* OR CPG-7909 OR CpG-oligonucleotide* OR Debio-1143 OR Deferipron* OR DHA-type-compound-9 OR Digoxin* OR Dilazep* OR Disulfiram* OR Droxinostat* OR Durvalumab* OR EK16A OR Euphoria-Kansui-extract* OR EXO-Tat* OR Flagellin* OR Givinostat* OR Gliotoxin* OR Gnidimacrin* OR GS986 OR Heme-arginat* OR Hexamethylene-bisacetamid* OR Hydroxyurea* OR I-BET OR I-BET-151 OR Imiquimod* OR Ionomycin* OR Ipilimumab* OR JQ1 OR Juglon* OR KAT5-Inhibitor* OR KD5170 OR LCL-161 OR M344 OR Maraviroc* OR MCB-613 OR Metacept* OR MG-132 OR MG-149 OR MGD-486 OR MGN-1703 OR MMQO OR MS-417 OR Nivolumab* OR ODN2006 OR ODN2040 OR OTX-015 OR Oxamflatin* OR Oxoglaucin* OR Pam2CSK4 OR Pam3CS OR Panobinostat* OR Pembrolizumab* OR PFI-1 OR PH01 OR PH02 OR PH03 OR PH04 OR PH05 OR Phythaemagglutinin* OR Phythemagglutinin* OR Phytohaemagglutinin* OR Phyto-haemagglutinin* OR Phytohemagglutinin* OR Phyto-hemagglutinin* OR Piceatannol* OR PIM6 OR Pimelic-diphenylamide-106 OR PKF050-638 OR Pochonin-B OR Pochonin-C OR Poly-ICLC OR PR-957 OR Pracinostat* OR Psammaplin-A* OR Pyrimethamine* OR Pyroxamid* OR Quinolin-8-ol-derivative* OR Quinolon* OR R848 OR Radicicol OR Ratjadon* OR Resveratrol* OR rGal9 OR Ro5-3335 OR Romidepsin* OR RVX-208 OR S1P1-agonist* OR Salubrinal* OR SB-216763 OR SBI-0637142 OR Scrpitaid* OR Sodium-butyrat* OR ST7612AA OR Sudemycin-D6 OR TatR5M4 OR Terreic-acid* OR Tideglusib* OR TLR3 OR TLR7 OR TLR9 OR Trapoxin-A* OR Trichostatin-A* OR UMB-136 OR Valproic-acid* OR Velcad* OR Vesatolimod* OR Vorinostat* OR AV6 OR CAP OR CLBL OR DPP OR HHODC OR HKLM OR LMB OR IL2 OR IL6 OR IL7 OR IL21 OR IL-2 OR IL-6 OR IL-7 OR IL-21 OR interleukin-2 OR interleukin-6 OR interleukin-7 OR interleukin-21 OR tumor-necrosis-factor* OR Tnf-alpha OR ALT-803 OR CYT-107):ab,ti,kw,de) AND ('virus latency'/de OR reservoir/de OR 'disease reservoir'/de OR (latenc* OR latent* OR reservoir* OR (shock NEAR/3 kill*)):Ab,ti))) NOT ('case report'/de OR 'case study'/de OR (case-report* OR case-stud* OR case-ser*):Ab,ti) NOT ([conference abstract]/lim) NOT ([animals]/lim NOT [humans]/lim)

## Medline ALL Ovid

(exp HIV/ OR exp HIV Infections/ OR Human immunodeficiency virus protein/ OR ((Human ADJ (immunodeficienc* OR immun*-deficienc*) ADJ virus*) OR hiv OR (acquired ADJ (immunodeficienc* OR immun*-deficienc*) ADJ syndrome*) OR aids).ab,ti.) AND (((Virus Latency /) AND (Virus Activation /)) OR (((Laten* OR reservoir*) ADJ3 (revers* OR reactivat* OR activat*)) OR (shock ADJ3 kill*)).ab,ti,kw. OR ((Histone Deacetylase Inhibitors/OR Interleukin-2/OR Interleukin-6/OR Interleukin-7/OR interleukin 21/ OR Tumor Necrosis Factor-alpha/OR ((Latency ADJ3 revers*) OR (shock ADJ3 kill*) OR ((hdac OR bromodomain* OR Bet) ADJ3 inhibit*) OR ((histone) ADJ3 (deacetylase OR methyltransferase OR methyl-transferase) ADJ6 inhibit*) OR ((DNA OR desoxyribonucle*) ADJ3 (methyltransferase OR methyl-transferase) ADJ6 inhibitor*) OR (BRG ADJ3 Associated-Factors ADJ3 Inhibitor*) OR deazaneplanocin-A* OR abexinostat* OR ACSS2-agonist* OR agatolimod* OR alobresib* OR alteminostat* OR apabetalon* OR apicidin* OR AZ391 OR azacitidin* OR Azacytidine* OR Azadeoxycytidin* OR belinostat* OR birabresib* OR BIX-01294 OR bromosporin* OR butyric-acid* OR cavrotolimod* OR CG05 OR CG06 OR Chaetocin* OR Chidamid* OR citarinostat* OR cobitolimod* OR cpg-10101 OR cxd-101 OR dacinostat* OR decitabin* OR depudecin* OR entinostat* OR entolimod* OR epetirimod* OR EPZ-6438 OR fimepinostat* OR flucytosine-deoxyribosid* OR gardiquimod* OR givinostat* OR glucopyranosyl-lipid-A* OR GSK-34 OR guadecitabin* OR guretolimod* OR hydralazin* OR imiquimod* OR inobrodib* OR isatoribin* OR ivaltinostat* OR JMF1080 OR lapretolimod* OR Largazoles* OR lefitolimod* OR lirametostat* OR litenimod* OR loxoribin* OR mivebresib* OR mocetinostat* OR molibresib* OR motolimod* OR phthaloyltryptophan* OR nanatinostat* OR oxamflatin* OR panobinostat* OR pelabresib* OR pinometostat* OR pivaloyloxymethyl-butyrat* OR pracinostat* OR quisinostat* OR rec-2282 OR remetinostat* OR resiquimod* OR resiquimod-pegol* OR resminostat* OR ricolinostat* OR rintatolimod* OR roducitabin* OR romidepsin* OR SDL148 OR SDL256 OR selgantolimod* OR sirtinol* OR Sodium-crotonat* OR sotirimod* OR tacedinalin* OR tazemetostat* OR tefinostat* OR telratolimod* OR Thiophenyl-benzamid* OR tributyrin* OR trichostatin-A* OR trotabresib* OR tubacin* OR tucidinostat* OR UNC-0638 OR UNC-1999 OR UNC-926 OR valemetostat* OR verpasep-caltespen* OR vesatolimod* OR vidutolimod* OR vorinostat* OR zebularin* OR 3-ingenol* OR 3M-002 OR 7SK-snRNP-disrupter* OR 8-azaguanin* OR ABX464 OR Abyssomicin-2 OR AG555 OR AGK2 OR Alpha-CD28 OR Alpha-CD3 OR Anti-CTLA-4 OR Anti-PD1 OR Apicidin* OR Aplysiatoxin* OR As2O3 OR Baf-inhibitor* OR Belinostat* OR Benzotriazole* OR Birinapant* OR BMS-936559 OR BRD3308 OR Bryolog* OR Bucladesin* OR Cardiac-glycoside-aglycone* OR Carfilzomib* OR Cemiplimab* OR CL413 OR Clomifen* OR CPG-7909 OR CpG-oligonucleotide* OR Debio-1143 OR Deferipron* OR DHA-type-compound-9 OR Digoxin* OR Dilazep* OR Disulfiram* OR Droxinostat* OR Durvalumab* OR EK16A OR Euphoria-Kansui-extract* OR EXO-Tat* OR Flagellin* OR Givinostat* OR Gliotoxin* OR Gnidimacrin* OR GS986 OR Heme-arginat* OR Hexamethylene-bisacetamid* OR Hydroxyurea* OR I-BET OR I-BET-151 OR Imiquimod* OR Ionomycin* OR Ipilimumab* OR JQ1 OR Juglon* OR KAT5-Inhibitor* OR KD5170 OR LCL-161 OR M344 OR Maraviroc* OR MCB-613 OR Metacept* OR MG-132 OR MG-149 OR MGD-486 OR MGN-1703 OR MMQO OR MS-417 OR Nivolumab* OR ODN2006 OR ODN2040 OR OTX-015 OR Oxamflatin* OR Oxoglaucin* OR Pam2CSK4 OR Pam3CS OR Panobinostat* OR Pembrolizumab* OR PFI-1 OR PH01 OR PH02 OR PH03 OR PH04 OR PH05 OR Phythaemagglutinin* OR Phythemagglutinin* OR Phytohaemagglutinin* OR Phyto-haemagglutinin* OR Phytohemagglutinin* OR Phyto-hemagglutinin* OR Piceatannol* OR PIM6 OR Pimelic-diphenylamide-106 OR PKF050-638 OR Pochonin-B OR Pochonin-C OR Poly-ICLC OR PR-957 OR Pracinostat* OR Psammaplin-A* OR Pyrimethamine* OR Pyroxamid* OR Quinolin-8-ol-derivative* OR Quinolon* OR R848 OR Radicicol OR Ratjadon* OR Resveratrol* OR rGal9 OR Ro5-3335 OR Romidepsin* OR RVX-208 OR S1P1-agonist* OR Salubrinal* OR SB-216763 OR SBI-0637142 OR Scrpitaid* OR Sodium-butyrat* OR ST7612AA OR Sudemycin-D6 OR TatR5M4 OR Terreic-acid* OR Tideglusib* OR TLR3 OR TLR7 OR TLR9 OR Trapoxin-A* OR Trichostatin-A* OR UMB-136 OR Valproic-acid* OR Velcad* OR Vesatolimod* OR Vorinostat* OR AV6 OR CAP OR CLBL OR DPP OR HHODC OR HKLM OR LMB OR IL2 OR IL6 OR IL7 OR IL21 OR IL-2 OR IL-6 OR IL-7 OR IL-21 OR interleukin-2 OR interleukin-6 OR interleukin-7 OR interleukin-21 OR tumor-necrosis-factor* OR Tnf-alpha OR ALT-803 OR CYT-107).ab,ti,kw.) AND (Virus Latency/OR Disease Reservoirs/OR (latenc* OR latent* OR reservoir* OR (shock ADJ3 kill*)).ab,ti.))) NOT (Case Reports/ OR (case-report* OR case-stud* OR case-ser*).ab,ti.) NOT (news OR congres* OR abstract* OR book* OR chapter* OR dissertation abstract*).pt. NOT (exp animals/ NOT humans/)

## Web of Science

TS=((((Human NEAR/1 (immunodeficienc* OR immun*-deficienc*) NEAR/1 virus*) OR hiv OR (acquired NEAR/1 (immunodeficienc* OR immun*-deficienc*) NEAR/1 syndrome*) OR aids)) AND ((((Laten* OR reservoir*) NEAR/2 (revers* OR reactivat* OR activat*)) OR (shock NEAR/2 kill*)) OR ((((Latency NEAR/2 revers*) OR (shock NEAR/2 kill*) OR ((hdac OR bromodomain* OR Bet) NEAR/2 inhibit*) OR ((histone) NEAR/2 (deacetylase OR methyltransferase OR methyl-transferase) NEAR/5 inhibit*) OR ((DNA OR desoxyribonucle*) NEAR/2 (methyltransferase OR methyl-transferase) NEAR/5 inhibitor*) OR (BRG NEAR/2 Associated-Factors NEAR/2 Inhibitor*) OR deazaneplanocin-A* OR abexinostat* OR ACSS2-agonist* OR agatolimod* OR alobresib* OR alteminostat* OR apabetalon* OR apicidin* OR AZ391 OR azacitidin* OR Azacytidine* OR Azadeoxycytidin* OR belinostat* OR birabresib* OR BIX-01294 OR bromosporin* OR butyric-acid* OR cavrotolimod* OR CG05 OR CG06 OR Chaetocin* OR Chidamid* OR citarinostat* OR cobitolimod* OR cpg-10101 OR cxd-101 OR dacinostat* OR decitabin* OR depudecin* OR entinostat* OR entolimod* OR epetirimod* OR EPZ-6438 OR fimepinostat* OR flucytosine-deoxyribosid* OR gardiquimod* OR givinostat* OR glucopyranosyl-lipid-A* OR GSK-34 OR guadecitabin* OR guretolimod* OR hydralazin* OR imiquimod* OR inobrodib* OR isatoribin* OR ivaltinostat* OR JMF1080 OR lapretolimod* OR Largazoles* OR lefitolimod* OR lirametostat* OR litenimod* OR loxoribin* OR mivebresib* OR mocetinostat* OR molibresib* OR motolimod* OR phthaloyltryptophan* OR nanatinostat* OR oxamflatin* OR panobinostat* OR pelabresib* OR pinometostat* OR pivaloyloxymethyl-butyrat* OR pracinostat* OR quisinostat* OR rec-2282 OR remetinostat* OR resiquimod* OR resiquimod-pegol* OR resminostat* OR ricolinostat* OR rintatolimod* OR roducitabin* OR romidepsin* OR SDL148 OR SDL256 OR selgantolimod* OR sirtinol* OR Sodium-crotonat* OR sotirimod* OR tacedinalin* OR tazemetostat* OR tefinostat* OR telratolimod* OR Thiophenyl-benzamid* OR tributyrin* OR trichostatin-A* OR trotabresib* OR tubacin* OR tucidinostat* OR UNC-0638 OR UNC-1999 OR UNC-926 OR valemetostat* OR verpasep-caltespen* OR vesatolimod* OR vidutolimod* OR vorinostat* OR zebularin* OR 3-ingenol* OR 3M-002 OR 7SK-snRNP-disrupter* OR 8-azaguanin* OR ABX464 OR Abyssomicin-2 OR AG555 OR AGK2 OR Alpha-CD28 OR Alpha-CD3 OR Anti-CTLA-4 OR Anti-PD1 OR Apicidin* OR Aplysiatoxin* OR As2O3 OR Baf-inhibitor* OR Belinostat* OR Benzotriazole* OR Birinapant* OR BMS-936559 OR BRD3308 OR Bryolog* OR Bucladesin* OR Cardiac-glycoside-aglycone* OR Carfilzomib* OR Cemiplimab* OR CL413 OR Clomifen* OR CPG-7909 OR CpG-oligonucleotide* OR Debio-1143 OR Deferipron* OR DHA-type-compound-9 OR Digoxin* OR Dilazep* OR Disulfiram* OR Droxinostat* OR Durvalumab* OR EK16A OR Euphoria-Kansui-extract* OR EXO-Tat* OR Flagellin* OR Givinostat* OR Gliotoxin* OR Gnidimacrin* OR GS986 OR Heme-arginat* OR Hexamethylene-bisacetamid* OR Hydroxyurea* OR I-BET OR I-BET-151 OR Imiquimod* OR Ionomycin* OR Ipilimumab* OR JQ1 OR Juglon* OR KAT5-Inhibitor* OR KD5170 OR LCL-161 OR M344 OR Maraviroc* OR MCB-613 OR Metacept* OR MG-132 OR MG-149 OR MGD-486 OR MGN-1703 OR MMQO OR MS-417 OR Nivolumab* OR ODN2006 OR ODN2040 OR OTX-015 OR Oxamflatin* OR Oxoglaucin* OR Pam2CSK4 OR Pam3CS OR Panobinostat* OR Pembrolizumab* OR PFI-1 OR PH01 OR PH02 OR PH03 OR PH04 OR PH05 OR Phythaemagglutinin* OR Phythemagglutinin* OR Phytohaemagglutinin* OR Phyto-haemagglutinin* OR Phytohemagglutinin* OR Phyto-hemagglutinin* OR Piceatannol* OR PIM6 OR Pimelic-diphenylamide-106 OR PKF050-638 OR Pochonin-B OR Pochonin-C OR Poly-ICLC OR PR-957 OR Pracinostat* OR Psammaplin-A* OR Pyrimethamine* OR Pyroxamid* OR Quinolin-8-ol-derivative* OR Quinolon* OR R848 OR Radicicol OR Ratjadon* OR Resveratrol* OR rGal9 OR Ro5-3335 OR Romidepsin* OR RVX-208 OR S1P1-agonist* OR Salubrinal* OR SB-216763 OR SBI-0637142 OR Scrpitaid* OR Sodium-butyrat* OR ST7612AA OR Sudemycin-D6 OR TatR5M4 OR Terreic-acid* OR Tideglusib* OR TLR3 OR TLR7 OR TLR9 OR Trapoxin-A* OR Trichostatin-A* OR UMB-136 OR Valproic-acid* OR Velcad* OR Vesatolimod* OR Vorinostat* OR AV6 OR CAP OR CLBL OR DPP OR HHODC OR HKLM OR LMB OR IL2 OR IL6 OR IL7 OR IL21 OR IL-2 OR IL-6 OR IL-7 OR IL-21 OR interleukin-2 OR interleukin-6 OR interleukin-7 OR interleukin-21 OR tumor-necrosis-factor* OR Tnf-alpha OR ALT-803 OR CYT-107)) AND ((latenc* OR latent* OR reservoir* OR (shock NEAR/2 kill*)))))) AND DT=(article) AND LA=(english)

## Cochrane CENTRAL

(((Human NEXT/1 (immunodeficienc* OR immun* NEXT deficienc*) NEXT/1 virus*) OR hiv OR (acquired NEXT/1 (immunodeficienc* OR immun* NEXT deficienc*) NEXT/1 syndrome*) OR aids):Ab,ti) AND ((latenc* OR latent* OR reservoir* OR (shock NEAR/3 kill*)):Ab,ti)
